# Cortical scaling of the neonatal brain in typical and altered development

**DOI:** 10.1073/pnas.2416423122

**Published:** 2025-04-08

**Authors:** Alexandra F. Bonthrone, Daniel Cromb, Andrew Chew, Barat Gal-Er, Christopher Kelly, Shona Falconer, Tomoki Arichi, Kuberan Pushparajah, John Simpson, Mary A. Rutherford, Joseph V. Hajnal, Chiara Nosarti, A. David Edwards, Jonathan O’Muircheartaigh, Serena J. Counsell

**Affiliations:** ^a^Centre for the Developing Brain, Research Department of Early Life Imaging, School of Biomedical Engineering and Imaging Sciences, King’s College London, London SE1 7EH, United Kingdom; ^b^Department of Paediatric Neurosciences, Evelina London Children’s Hospital, London SE1 7EH, United Kingdom; ^c^Medical Research Council Centre for Neurodevelopmental Disorders, King’s College London, London SE1 1UL, United Kingdom; ^d^Research Department of Cardiovascular Imaging, School of Biomedical Engineering & Imaging Sciences, King’s College London, London SE1 7EH, United Kingdom; ^e^Department of Fetal and Paediatric Cardiology, Evelina London Children’s Hospital, London SE1 7EH, United Kingdom; ^f^Department of Child and Adolescent Psychiatry, Institute of Psychiatry, Psychology and Neuroscience, King’s College London, London SE5 8AB, United Kingdom; ^g^Department of Forensic and Neurodevelopmental Sciences, Institute of Psychiatry, Psychology and Neuroscience, King’s College London, London SE5 8AB, United Kingdom

**Keywords:** cortical folding, neonatal brain, MRI, allometric scaling

## Abstract

Theoretically derived scaling laws capture the nonlinear relationships between rapidly expanding brain volume and cortical gyrification across mammalian species and in adult humans. However, the preservation of these laws has not been comprehensively assessed in typical or pathological brain development. Here, we assessed the scaling laws governing cortical thickness (CT), surface area (SA), and cortical folding in the neonatal brain. We also assessed multivariate morphological terms that capture brain size, shape, and folding processes. The sample consisted of 345 typically developing infants, 73 preterm infants, and 107 infants with congenital heart disease (CHD) who underwent brain MRI. Our results show that typically developing neonates and those with CHD follow the cortical folding scaling law obtained from mammalian brains, children, and adults which captures the relationship between exposed SA, total SA, and CT. Cortical folding scaling was not affected by gestational age at birth, postmenstrual age at scan, sex, or multiple birth in these populations. CHD was characterized by a unique reduction in the multivariate morphological term capturing size, suggesting that CHD affects cortical growth overall but not cortical folding processes. In contrast, preterm birth was characterized by altered cortical folding scaling and altered shape, suggesting that the developmentally programmed processes of cortical folding are disrupted in this population. The degree of altered shape was associated with cognitive abilities in early childhood in preterm infants.

## Significance

The mammalian, adult human, and child cortices fold according to a universal scaling law that relates exposed surface area (SA) to total SA and cortical thickness. We demonstrate that this law also applies to typically developing neonates. We report that although the cortex is smaller, cortical scaling is preserved in infants with congenital heart disease, suggesting that this fundamental brain property is robust to the effects of reduced oxygen and nutrient delivery in utero. In contrast, this scaling law is altered in preterm birth, suggesting that early exposure to the extrauterine environment disrupts cortical folding processes. The degree of altered shape of the cortex in preterm infants predicted cognition in early childhood.

Rapid cortical volumetric expansion and gyrification take place during the third trimester of human pregnancy and early postnatal life (1, 2). The dynamics of cortical development can be captured in two morphological measures, cortical thickness (CT) and surface area (SA), which individually have been found to have distinct spatiotemporal developmental patterns (3, 4), genetic influences (5), and signatures in neurodevelopmental conditions (6–8). However, the development of CT and SA is linked to both volumetric growth and cortical folding processes. Allometric scaling models employ logarithmic transformation to measure scaling coefficients (α) of nonlinear relationships between anatomical brain characteristics in adults (9, 10) and across mammalian species (11, 12). However, the allometric scaling principles of SA, CT, and cortical folding in the typical neonatal brain, and alterations associated with adverse neurodevelopmental conditions, have yet to be fully investigated.

Previous studies investigating the allometric scaling relationships between total SA and volume [log_10_(total SA) ~ α*log_10_(supratentorial volume)] in preterm neonates scanned from birth (25 to 28 wk) to 48 wk postmenstrual age at scan (PMA) report a total SA to supratentorial volume scaling coefficient of 1.27 to 1.29 (13, 14). Importantly, these studies comprised preterm infants and assessed a wide PMA range, and therefore, it is not possible to disentangle typical brain development from the sequelae of preterm birth.

Another universal model describes the scaling law governing cortical folding in mammalian brains (11) and across children and adult humans (15) by capturing relationships between CT, total SA, and exposed gyral SA (Total SA*CT^0.5^ ~ exposed SA^1.25^, model details in methods). In this model, changes in one morphological characteristic are necessarily accompanied by compensatory alterations in another, suggesting the dynamics of cortical development may be better assessed with multivariate variables capturing the relationships between total SA, exposed SA, and CT, rather than individual cortical metrics. These terms, developed by Wang and colleagues (16), are orthogonal vectors defined based on the universal scaling law (Total SA*CT^0.5^ ~ exposed SA^1.25^; *SI Appendix*, Fig. S1): i) an offset term, representing orthogonal deviance from the 3D plane captured by the scaling law, characterizing the cellular, molecular, and mechanical factors which encourage the cortical surface to fold, it increases with increasing total SA and CT and decreasing exposed SA; ii) an isometric term capturing size changes, which increases with total SA, exposed SA, and CT; and iii) a shape term carrying additional information about shape which increases with higher total and exposed SA, but lower CT.

Allometric scaling or multivariate morphometric terms in the typical neonatal brain and in altered neurodevelopmental conditions at around the time of typical delivery (≥37+0 wk PMA) have not been comprehensively characterised. The first aim of this study was to characterize the allometric scaling coefficients governing total SA, CT, and cortical folding as well as multivariate morphological terms (offset, isometric, and shape) and associations with sex, gestational age at birth (GA), PMA, and multiple birth in the typical neonatal brain using MRI data collected from the developing human connectome project (dHCP) (17). The second aim was to assess how exposure to adverse developmental conditions alters cortical scaling or multivariate morphology, focusing on two distinct populations: infants born preterm (infants in dHCP born <33+0 wk GA) and infants with congenital heart disease (CHD) requiring surgery within the first year after birth [imaged using the same scanner and acquisitions as dHCP (18)]. The final aim was to investigate whether allometric scaling or multivariate morphology is associated with early childhood neurodevelopmental outcomes in typical and clinical populations.

## Results

T2-weighted neonatal brain MR images were acquired at 37 to 44+6 wk PMA from 345 healthy control infants delivered at term, 73 preterm infants, and 107 infants with CHD without major lesions (*SI Appendix*, Table S1 contains demographics). Images were processed using the dHCP neonatal cortical surface reconstruction pipeline (19) to provide measures of total SA, exposed SA, mean curvature corrected CT, and supratentorial brain volume. Analyses were conducted in R v.4.4.0 using linear regressions with permutation testing (n = 5,000).

### Scaling Coefficients and Multivariate Morphology in Typical and Altered Development.

In typically developing infants, the cortical folding scaling coefficient (α [95% CI] 1.26 [1.21 to 1.30]) was not significantly different from the adult and cross-species coefficient of 1.25 (*P* = 0.719). The total SA scaling coefficient (0.873 [0.830 to 0.914]) was significantly different from the theoretical coefficient of 2/3 but did not differ significantly from the coefficient previously observed in a normative adult sample (α = 0.89, *P* = 0.418) (9). In contrast, the CT scaling coefficient (0.087 [0.040 to 0.132]) significantly differed from both the theoretical (1/3, *P* < 0.001) and previously observed adult (0.03, *P* = 0.017) coefficients (9).

**Fig. 1.**
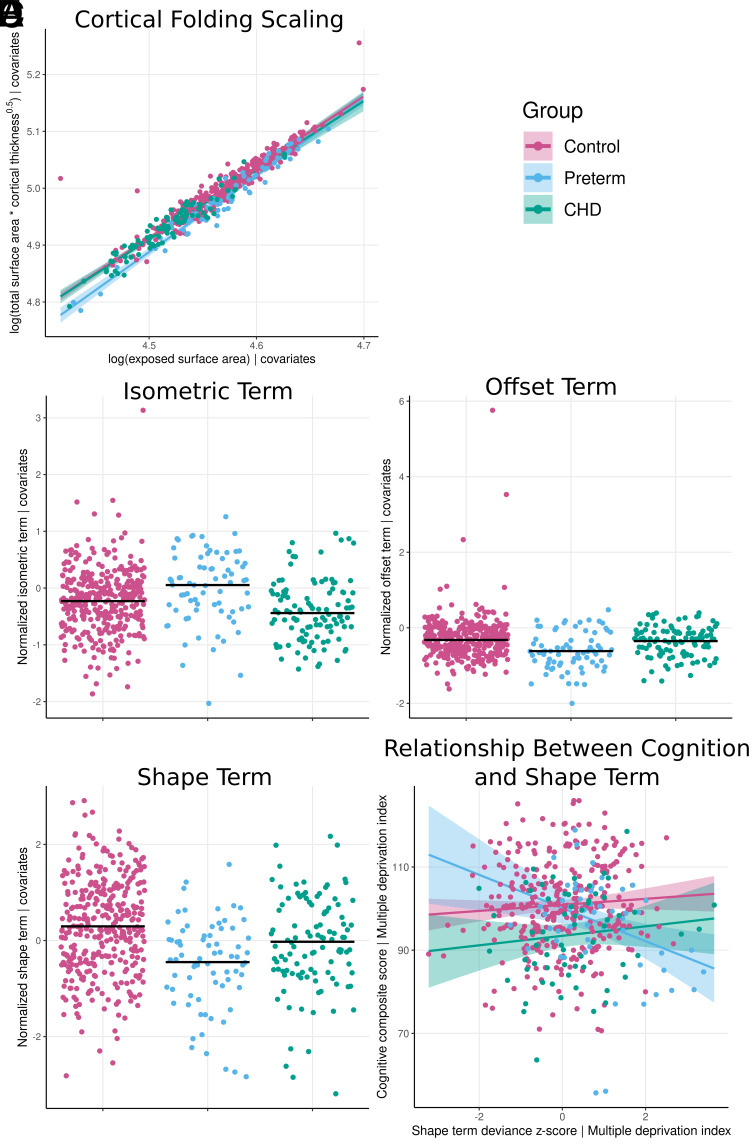
Differences in (*A*) cortical folding scaling and multivariate morphological terms (*B*–*D*) between groups adjusting for PMA, PMA^2^, sex, birth weight z-score, and multiple birth. Variables in *B*–*D* were z-scored to controls for visualization purposes as terms are unitless. (*E*) Association between multivariate shape term deviance z-score and cognitive composite score across each group adjusting for socioeconomic status.

The preterm allometric scaling coefficient for cortical folding (α [95% CI] 1.41 [1.30 to 1.51]) was significantly different from controls and infants with CHD (Table 1 and Fig. 1*A*). The cortical folding scaling coefficient in CHD (1.18 [1.08 to 1.28]) was not significantly different from controls (Table 1).

**Table 1.** The effect of group on cortical allometric scaling relationships

Model	B (95% CI)	T	P (p_FWE_)
Typically developing controls (reference) and preterm infants			
*Allometric scaling coefficients*			
Total SA	0.030 (−0.044 to 0.103)	0.790	0.441 (0.882)
CT	0.009 (−0.062 to 0.080)	0.248	0.802 (0.882)
Cortical folding	0.155 (0.050 to 0.259)	2.92	0.005 (0.020)*
*Multivariate morphological terms*			
Offset	−0.012 (−0.018 to −0.007)	−4.39	<0.001 (<0.001)*
Isometric	0.035 (0.007 to 0.062)	2.48	0.014 (0.042)*
Shape	−0.141 (−0.190 to −0.092)	−5.75	<0.001 (<0.001)*
Typically developing controls (reference) and infants with CHD			
*Allometric scaling coefficients*			
Total SA	−0.001 (−0.080 to 0.077)	−0.036	0.970 (1.00)
CT	−0.016 (−0.093 to −0.061)	−0.409	0.676 (1.00)
Cortical folding	0.111 (−0.004 to 0.227)	1.90	0.060 (0.240)
*Multivariate morphological terms*			
Offset	−0.004 (−0.009 to <0.001)	−1.65	0.102 (0.306)
Isometric	−0.047 (−0.071 to −0.023)	−3.88	<0.001 (0.001)*
Shape	−0.051 (−0.098 to −0.004)	−2.11	0.039 (0.195)
Preterm infants (reference) and infants with CHD			
*Allometric scaling coefficients*			
Total SA	−0.018 (−0.108 to 0.071)	−0.407	0.682 (0.682)
CT	−0.073 (−0.164 to 0.017)	−1.59	0.112 (0.224)
Cortical folding	−0.145 (−0.258 to −0.031)	−2.52	0.012 (0.048)*
*Multivariate morphological terms*			
Offset	0.012 (0.005 to 0.018)	3.62	<0.001 (0.004)*
Isometric	−0.062 (−0.102 to −0.022)	−3.05	0.002 (0.010)*
Shape	0.078 (0.007 to 0.149)	2.18	0.031 (0.093)

^*^Significant p_FWE_ < 0.05; model covariates included PMA, PMA^2^, sex, birth weight z-score, and multiple birth. GA also included in model with CHD and typically developing controls.

The allometric scaling coefficients for total SA (α [95% CI] preterm 0.893 [0.820 to 0.968], CHD 0.855 [0.770 to 0.940]) and CT (preterm 0.079 [0.020 to 0.138], CHD 0.095 [0.090 to 0.171]) were not significantly different from controls (Table 1).

Infants with CHD had lower isometric terms compared to controls and preterm infants (Table 1 and Fig. 1*B*). Preterm infants had significantly altered offset and shape terms compared to controls (Table 1 and Fig. 1 *C* and *D*). Offset terms were also significantly different between preterm infants and infants with CHD (Table 1).

GA was associated with multivariate isometric terms in controls (0.030 [0.041 to −0.019], t = −5.30, pFWE ≤ 0.001) and preterm infants (0.017 [0.009 to 0.026], t = 4.11, pFWE = 0.001). GA was also associated with the shape term in controls (0.040 [0.019 to 0.061], t = 3.78, pFWE < 0.001), and the offset term in preterm infants (0.003 [0.002 to 0.004], t = 4.27, pFWE < 0.001) (see *SI Appendix*, Fig. S2 for plots).

Birth weight z-score was associated with multivariate isometric terms across all groups (*SI Appendix*, Tables S2–S4, pFWE ≤ 0.002). Birth weight z-score was also associated with offset terms (0.004 [0.001-0.006], t = 3.02, pFWE = 0.024) in typically developing controls (see *SI Appendix*, Fig. S3 for plots).

The isometric term was also associated with sex in typically developing controls (0.027 [0.007 to 0.047], t = 2.63, *P* = 0.040, *SI Appendix*, Fig. S4), and PMA and PMA^2^ in preterm infants. There were no other significant relationships between multivariate morphology and demographics (pFWE > 0.05, *SI Appendix*, Tables S2–S4).

In preterm infants, days of parenteral nutrition and days of respiratory support were not associated with allometric scaling coefficients or multivariate terms (pFWE > 0.05, *SI Appendix*, Table S5). In infants with CHD, multivariate isometric terms were significantly higher in left-sided lesions compared to abnormal streaming (0.081[0.041 to 0.121], t = 4.03, pFWE < 0.001) and right-sided lesions (–0.360[–0.657 to –0.064], t = –2.38, *P* = 0.021, *SI Appendix*, Fig. S5*A*). Higher isometric terms were also associated with cerebral oxygen delivery (<0.001 [<0.001 to <0.001], t = 3.54, pFWE = 0.020, *SI Appendix*, Fig. S5*B*).

### Multivariate Shape Is Associated with Early Childhood Cognitive Abilities in Preterm Infants.

All controls, 57 preterm infants, and 65 infants with CHD underwent a Bayley scales of infant and toddler development-3rd edition (20) assessment at 17.26 to 40.29 mo corrected age providing cognitive, language, and motor composite scores.

An individual’s degree of deviation from the typical population across each scaling metric and multivariate morphological term was calculated as previously reported (9). A complete linear model for each metric was constructed for the typically developing control population and then applied to infants with CHD and preterm infants. Residuals were extracted and z-scored to the controls to measure an individual’s deviance from the typical population, given their supratentorial volume/exposed SA (for scaling relationships), PMA, PMA^2^, sex, and multiple birth status.

There were no significant associations between deviance z-scores and neurodevelopmental outcomes in typically developing infants (pFWE > 0.05, *SI Appendix*, Table S6). There was a significant interaction between group and shape term deviance z-score when predicting cognitive composite scores (Table 2 and Fig. 1*E*). The z-score predicted cognitive scores in preterm infants (*P* = 0.001, bootstrapped R^2^ for shape z-score = 0.115), but not controls (*P* = 0.222) or CHD (*P* = 0.225). Post hoc analysis revealed the interaction remained significant when excluding controls (*P* < 0.001). There were no other significant interactions between deviance z-scores and neurodevelopmental outcomes (Table 2).

**Table 2.** Interactions between group and scaling relationship/multivariate morphological deviance z-scores when predicting outcomes

	B (95% CI)	t	p (pFWE)
Typically developing controls (reference) vs preterm infants			
Cognitive Composite Scores			
Total SA scaling	−2.87 (−6.05 to 0.323)	−1.77	0.076 (0.762)
CT scaling	3.90 (0.776 to 7.02)	2.45	0.015 (0.213)
Cortical Folding scaling	−3.92 (−7.40 to 0.353)	−2.16	0.031 (0.400)
Offset term	−0.79 (−4.21 to 2.62)	−0.457	0.659 (1.00)
Isometric term	0.029 (−3.03 to 3.08)	0.019	0.985 (1.00)
Shape term	−4.78 (−7.83 to −1.73)	−3.08	0.002 (0.036)*
Language Composite Scores			
Total SA scaling	−3.19 (−7.63 to 1.25)	−1.41	0.164 (1.00)
CT scaling	6.37 (1.78 to 11.0)	2.73	0.006 (0.102)
Cortical Folding scaling	−5.00 (−9.97 to −0.040)	−1.98	0.048 (0.578)
Offset term	−0.450 (−5.33 to 4.43)	−0.181	0.863 (1.00)
Isometric term	3.49 (−1.03 to 8.00)	1.52	0.133 (1.00)
Shape term	−6.06 (−10.5 to −1.65)	−2.70	0.008 (0.123)
Motor Composite Scores			
Total SA scaling	−1.78 (−4.70 to 1.14)	−1.20	0.220 (1.00)
CT scaling	1.47 (−1.40 to 4.34)	1.01	0.304 (1.00)
Cortical Folding scaling	−4.72 (−7.97 to −1.46)	−2.85	0.007 (0.106)
Offset term	−2.95 (−6.07 to 0.173)	−1.86	0.062 (0.684)
Isometric term	0.217 (−2.57 to 3.01)	0.153	0.881 (1.00)
Shape term	−1.80 (−4.61 to 1.00)	−1.26	0.213 (1.00)
Typically developing controls (reference) vs infants with CHD			
Cognitive Composite Scores			
Total SA scaling	−1.26 (−4.16 to 1.63)	−0.857	0.401 (1.00)
CT scaling	−1.30 (−4.07 to 1.48)	−0.919	0.355 (1.00)
Cortical Folding scaling	−2.37 (−5.71 to 0.984)	−1.39	0.164 (1.00)
Offset term	−1.73 (−4.75 to 1.28)	−1.13	0.262 (1.00)
Isometric term	−4.35 (−8.20 to −0.492)	−2.22	0.025 (0.457)
Shape term	0.783 (−1.89 to 3.45)	0.576	0.575 (1.00)
Language Composite Scores			
Total SA scaling	−1.48 (−5.82 to 2.86)	−0.671	0.508 (1.00)
CT scaling	−3.47 (−8.62 to 1.67)	−1.33	0.185 (1.00)
Cortical Folding scaling	−2.86 (−7.42 to 1.70)	−1.23	0.214 (1.00)
Offset term	−5.23 (−10.9 to 0.476)	−1.80	0.070 (1.00)
Isometric term	−1.84 (−6.53 to 2.86)	−0.769	0.438 (1.00)
Shape term	0.920 (−3.24 to 5.078)	0.435	0.659 (1.00)
Motor Composite Scores			
Total SA scaling	−0.262 (−2.94 to 2.41)	−0.192	0.855 (1.00)
CT scaling	−0.391 (−2.94 to 2.16)	−0.301	0.758 (1.00)
Cortical Folding scaling	−0.528 (−3.59 to 2.54)	−0.338	0.735 (1.00)
Offset term	−1.26 (−4.79 to 2.27)	−0.699	0.493 (1.00)
Isometric term	−0.158 (−2.91 to 2.59)	−0.113	0.919 (1.00)
Shape term	0.377 (−2.09 to 2.84)	0.301	0.769 (1.00)

^*^Significant results p_FWE_ <0.05; linear regression with 5,000 permutations, model covariate included index of multiple deprivation. Additional covariate for language models was if a parent’s first language was not English.

### Assessing Individual Cortical Metrics.

The effect of group on total SA, CT, gyrification index (total SA/exposed SA; GI), and supratentorial brain volume was assessed (*SI Appendix*, Table S7). Preterm infants had significantly lower SA, and GI, and higher CT compared to controls (pFWE ≤ 0.002). Infants with CHD had lower total cortical SA, GI, and supratentorial brain volume (pFWE ≤ 0.015). GI and CT were significantly lower in infants with CHD compared to preterm infants (pFWE ≤ 0.015). CT was associated with language scores in preterm infants (bootstrapped R^2^ = 0.027). There were no other associations between outcome measures and individual cortical metrics (*SI Appendix*, Table S8).

## Discussion

In this study, we demonstrate that typical neonatal cortical development conforms to the intrinsic principle folding scaling law that applies to child and adult human and other mammalian brains. Following preterm birth, but not CHD, the folding scaling coefficient was significantly different from the control population. Preterm birth was also characterized by reduced multivariate morphological shape and offset terms, while CHD was characterized by reduced isometric terms with intact offset and shape characteristics. These data suggest distinct cortical developmental changes in different at-risk populations. Scaling coefficients and multivariate morphology were not associated with neurodevelopmental outcomes in typical infants or those with CHD. In contrast, for a given preterm infant, the shape term’s degree of deviation from the typical population was associated with later cognition in early childhood. Finally, there were no differences in scaling principles of total SA and CT in the clinical cohorts.

The typically developing neonatal brain follows the same cortical folding scaling law as typical child and adult brains (15) and other mammalian cortices (11). The folding scaling coefficient may therefore represent information about the molecular, cellular, and mechanical mechanisms that shape mammalian cortical folding (21). The allometric scaling coefficient linking SA and supratentorial volume in typically developing neonates is significantly higher than the theoretical geometric scaling coefficient of 0.67, but not significantly different from the coefficient previously reported in the typical adult population (9). These results suggest that in the brains of typical infants born around the expected date of delivery (≥37 wk), as in the typical adult brain (22), higher volume is accompanied by a larger increase in SA, reflecting increased gyrification. The scaling coefficient between CT and volume, while significantly lower than the theoretical coefficient, was significantly higher than that reported in typical adults (9). In typical brain development, CT increases rapidly to a peak around 2 y before declining across adolescence and adulthood, while cerebral volume and SA increase into adolescence (4), which may explain the difference in scaling relationships. Together, our results suggest that, at the time of normal delivery, the typically developing brain follows the same scaling relationships for cortical folding and SA as adults which may reflect biologically conserved folding mechanisms. In contrast, the scaling coefficient for CT in infants was significantly higher than in adults, perhaps reflecting the unique developmental trajectory of CT in early childhood.

In preterm infants, the scaling coefficient for cortical folding is significantly different from the corresponding value in typically developing controls and the theoretically predicted value. The multivariate morphological term capturing factors encouraging folding (offset) was also significantly reduced in preterm infants compared to controls. The neurobiological mechanisms of altered cortical folding in premature infants are unclear. Reduced folding may be related to altered underlying white matter connections in preterm infants (23, 24). The scaling results suggest that altered cortical development following preterm birth in the absence of major destructive lesions is not due to fewer cortical neurons (11, 25). We identified lower supratentorial brain volume, total SA, and GI but thicker cortex in preterm infants compared to controls, a finding supported by previous studies (6, 26). Additionally, histological research suggests preterm birth alters neuronal maturation but is not associated with large-scale neuronal loss (27). Furthermore, adults born preterm show altered cortical folding scaling coefficients and multivariate morphological offset terms compared to controls (28), suggesting preterm birth alters developmentally programmed folding principles with effects across the lifespan.

The mechanisms behind the development of cortical folds are not well understood and likely encompass biomechanical and intrinsic cellular mechanisms. Barresi and colleagues (29) provide a comprehensive review of theories of cortical gyrification. Biomechanical forces may encompass tension from axons connecting cortical regions and the nonuniform expansion of cortical layers causing the formation of gyri. Other theories suggest that cortical folding is driven by differential growth and expansion of certain cell populations in the developing cortex, such as the apical radial glial cells in the ventricular zone, basal intermediate progenitor cells in the subventricular zone, and basal radial glial cells in the outer subventricular zone. The results of our study do not provide direct insight into the mechanisms underlying the development of gyrification. However, future research encompassing animal models and histopathological studies of the developing brain in CHD and premature birth using allometric scaling relationships as outcome measures may provide unique opportunities to characterize the cellular and biomechanical forces which drive cortical folding.

We also find the multivariate shape term is lower in infants born preterm compared to controls. Lower shape terms are associated with lower exposed and total SA but higher CT, and infants born preterm had higher CT and lower total SA and GI (total SA/exposed SA) compared to controls. Lower shape terms were associated with higher cognitive abilities in early childhood in preterm infants. It is possible that changes in the dynamics of CT and SA development, affecting the shape term, are adaptive processes in preterm infants which support cognitive development. Indeed, changes in the shape term were not associated with days of parenteral nutrition and respiratory support. Previous research in preterm infants has identified associations between cognition in early childhood and regional changes in shape characteristics such as CT (29), sulcal depth (30), and cortical curvature 31, 32 at the same timepoint as our study. These effects may have long-lasting implications, as total SA growth from 22 to 44 wk PMA has also been found to be associated with cognitive abilities in middle childhood in preterm infants (7).

Two previous studies reported the scaling coefficient between total cortical SA and volume in preterm infants was 1.27 to 1.29, significantly higher than the values reported in our study (13, 14). However, it is important to note that infants in previous studies were scanned from birth (22 to 30 wk) to 45 to 48 wk PMA, whereas our analyses were restricted to infants scanned at term equivalent age only. A small study comparing infants born preterm to fetuses imaged from 21 to 36 wk GA suggests allometric scaling between SA and brain volume is significantly higher in preterm infants; however, direct comparison between fetal and neonatal MR data remains challenging (33). This may reflect a different developmental period, as gyrification increases rapidly from around 28 wk GA (1, 2). Kapellou and colleagues previously reported lower GA and male sex are associated larger alterations in cortical SA scaling in preterm infants (13), which we did not replicate. The effect of sex and dose-dependent effects of prematurity may be stronger earlier in cortical development. Future research across the fetal and early preterm period is required to fully characterize allometric scaling across development.

In contrast to preterm infants, infants with CHD had significantly lower multivariate isometric terms than controls, reflecting smaller CT, total SA, and exposed SA, but showed no significant differences in scaling coefficients, offset or shape terms. Lower isometric terms were associated with reduced cerebral oxygen delivery in this population. CHD is associated with chronic suboptimal supply of oxygen, glucose, and other nutrients that support fetal and neonatal brain development (34–36). In keeping with this, several studies have identified reduced brain volumes in fetuses and neonates with CHD compared with healthy infants (37–40) the degree of which was associated with reduced cerebral substrate delivery (18, 34, 40). In our study, isometric terms were higher in infants with left-sided lesions compared to abnormal streaming of blood and right-sided lesions. However, in our study, 71% of left-sided lesions were aortic arch anomalies, which are expected to have mild effects of cerebral substrate delivery in the fetus (39). Given the sample size, it was not possible to divide infants with CHD further into individual diagnoses.

Reduced cortical folding has also been reported in infants with CHD (18, 41, 42); however, Cromb et al. (43) demonstrated that differences in GI between infants with CHD and controls were no longer significant when adjusting for supratentorial brain volume. We support these findings by demonstrating that the allometric scaling of cortical folding is not significantly different from either typically developing infants or the cross-species coefficient (11). The primary cerebral phenotype in CHD may therefore be smaller brain volumes, with proportionate reductions in cortical folding, rather than specifically disrupted folding mechanisms.

A strength of the approach of considering scaling coefficients and multivariate morphology, which capture different relationships between CT, total SA, and exposed SA, is that it considers the relationship between measures, rather than changes in individual metrics. When assessing individual cortical metrics, it is possible to conclude that infants with CHD and preterm infants have similar cerebral changes, reflected in reductions in volume, total SA, and GI compared to controls. However, allometric scaling relationships and multivariate morphology demonstrate distinct alterations, with CHD showing reduced brain size while preterm infants show aberrant cortical folding and shape compared to controls. These methods therefore distinguish between proportionate size reductions as compared to disproportionate reductions in total SA and GI and increases in CT which change the shape of the cortex. Further evidence that the differences in brain development are distinct in CHD and preterm infants can be seen in the differing patterns of cortical microstructural changes reported in these groups, with preterm infants displaying widespread reductions in neurite density index and increases in mean diffusivity (6), while infants with CHD show reductions in fractional anisotropy and orientation dispersion index and no differences in neurite density or mean diffusivity (44). Our work highlights the value of considering multiple covarying morphological variables when distinguishing phenotypes between clinical groups.

Our results have some limitations which are important to acknowledge. We used whole brain measures to assess cortical morphology; however, there may be important differences in regional scaling which would not be captured in this study. In this study, we chose to assess global scaling relationships given the rapid global brain growth seen in the neonatal period, and the global changes in brain size and folding previously reported in neonates with CHD and preterm infants. The dHCP dataset makes it possible to consider future analyses of scaling differences using regional and vertex-wise methods (17). In addition, our analysis assessed infants from 37+0 to 44+6 PMA; however, the dynamics of cortical scaling are likely different across different development periods. Future research should assess morphological development across the fetal and preterm period. In addition, the differences observed in this study are smaller than those reported in adult clinical populations by Wang and colleagues (16). Future studies assessing adult survivors of CHD and preterm birth could assess whether the differences observed in our study increase or diminish with further brain growth into childhood and adulthood.

In summary, the typical neonatal brain conforms to the scaling law governing cortical folding in children, adults, and other mammalian brains. We report distinct cortical developmental changes in different at-risk populations. Brains were smaller in infants with CHD, while preterm infants demonstrated altered cortical folding and brain shape compared to controls. Shape characteristics were associated with early childhood cognition in preterm infants but not controls or CHD.

## Materials and Methods

### Ethical Approval.

The National Research Ethics Service provided ethical approval (CHD: 07/H0707/105, 21/WA/0075; dHCP 14/LO/1169). In accordance with the Declaration of Helsinki, informed written parental consent was obtained before neonatal MRI and neurodevelopmental assessment.

### Recruitment.

Data from controls and preterm infants were from the dHCP 3rd neonatal data release (17). The control sample included 345 infants. Exclusion criteria were GA <37 wk, admission to the neonatal intensive care unit, a 1st-degree relative with a diagnosed neurodevelopmental condition (45), a neurodevelopmental outcome score below 70 (<2 SD from the test mean), and no evidence of walking at neurodevelopmental assessment.

The preterm sample consisted of 73 infants born 23 to 32+6 wk GA. Exclusion criteria included no brain MRI at ≥37 wk PMA and suspected/confirmed chromosomal abnormality/congenital syndrome. Information about days of parenteral nutrition and respiratory support with continuous positive airway pressure or invasive mechanical ventilation was extracted from clinical records.

Infants with CHD requiring cardiac catheterization or surgery before 1 y postnatal age (46) were recruited as fetuses or neonates from St Thomas’ Hospital, London October 2015-July 2023. Exclusion criteria were birth <37 wk GA, suspected/confirmed chromosomal abnormality/congenital syndrome, previous neonatal surgery (excluding cardiac catheterization), or suspected congenital infection (18). Infants with CHD were categorized as having cardiac lesions causing abnormal streaming of blood, left-sided, and right-sided lesions (38).

Exclusion criteria for all infants were no/motion corrupted neonatal brain MRI, major lesions on MRI (e.g. arterial ischemic stroke, parenchymal hemorrhage, >10 foci of punctate white matter injury) after review by two perinatal neuroradiologists, and failed surface reconstruction [either reconstruction did not complete or inaccurate surface reconstruction on visual inspection; 14 control infants excluded (3.9%), 3 CHD (2.7%), and 2 preterm infants (2.7%)].

### MRI Acquisition.

Brain MRI was performed during natural sleep following feeding on a Philips Achieva (Best, Netherlands) 3 Tesla system situated in the neonatal intensive care unit at St. Thomas’ Hospital using a 32-channel neonatal head coil and neonatal positioning device (Rapid Biomedical GmbH, Rimpar DE) (47). Hearing protection included silicone-based putty earplugs placed in the external auditory meatus (President Putty), neonatal earmuffs (MiniMuffs), and an acoustic hood placed over the infant. A pediatrician or nurse trained in MR procedures supervised scanning and monitored pulse oximetry, axillary temperature, electrocardiography, and respiratory rate throughout.

T2-weighted multislice turbo spin echo scans were acquired in sagittal and axial planes [repetition time (TR)/echo time (TE) 12,000/156 ms; flip angle 90°; slice thickness 1.6 mm; slice overlap 0.8 mm; in-plane resolution 0.8 × 0.8 mm; SENSE factor 2.11/2.58 (axial/sagittal)] with a 5 s noise ramp-up to avoid a startle response. T2-weighted volumes were reconstructed using a dedicated algorithm (reconstructed voxel size = 0.5 mm^3^) encompassing motion correction (48).

### MRI Processing.

T2-weighted brain MRI was processed using the dHCP structural pipeline (19). T2-weighted images were corrected for bias-field inhomogeneities, brain extracted and segmented into nine tissue classes using the Draw-EM algorithm (49). Supratentorial brain volume was calculated by summing cortical white matter, cortical gray matter, hippocampus and amygdala, and deep gray matter volumes. Left and right white, pial, and mid-thickness surfaces were extracted and inflated. All surface reconstructions were visually inspected. CT (curvature regressed out) and total pial SA were calculated and extracted excluding the midline. The area of the exposed pial cortex was calculated using the medical image registration toolkit (https://mirtk.github.io/) and connectome workbench v.1.5.0. An example of a pial cortex and exposed pial cortex reconstruction can be seen in *SI Appendix*, Fig. S6. Histograms of CT, total SA, and exposed SA can be seen in *SI Appendix*, Fig. S7.

### Neurodevelopmental Assessment.

Infants were invited to attend a neurodevelopmental follow-up assessment as close to 18 mo (dHCP) or 22 mo (CHD) corrected age as possible. The Bayley Scales of Infant and Toddler Development-3rd Edition (20) were administered by a developmental pediatrician or psychologist to obtain cognitive, language, and motor composite scores [test mean (SD) 100(15)]. In addition, parents completed a questionnaire which asked whether English was the first language of the child’s parents. Demographic differences between infants who attended follow-up compared to those who did not are noted in *SI Appendix*, Table S9.

### Socioeconomic Status.

IMD was calculated using the 2019 data release for postcode at birth and reported as ranks and quintiles (most to least deprived). IMD is a composite measure of socioeconomic status in England encompassing factors including income, employment, education, health, and crime.

### Cerebral Oxygen Delivery.

Cerebral oxygen delivery was measured in 85 infants with CHD using previously published methods (18), details of which can be found in supplement.

### Birth Weight z-Score.

Birth weight z-scores were calculated for all infants using the UK-WHO 1990 normative data from 23 wk GA.

### Statistical Analysis.

All analyses were conducted in R v.4.4.0. All demographic variables were presented with median and interquartile ranges or counts.

### Allometric Scaling.

The geometric scaling laws governing surface thickness, area, and total volume in three-dimensional objects of identical shape but different size are

[1] $$\mathrm{object}\mathrm{SA}∼k^{*}\mathrm{object}{\mathrm{volume}}^{2/3},$$

[2] $$\mathrm{object}\mathrm{surface}\mathrm{thickness}∼k^{*}\mathrm{object}{\mathrm{volume}}^{1/3}.$$

In these equations, k represents a constant, and 2/3 and 1/3 represent the scaling exponents (α). By logarithmically transforming these equations, α can be estimated. Linear regressions capturing the relationship between total SA and supratentorial volume Eq. **3** and CT and supratentorial volume Eq. **4** were calculated adjusting for sex, PMA, PMA^2^, and multiple birth.

[3] $$\log_{10}(\mathrm{total}\mathrm{SA})∼\alpha^{*}\log_{10}(\mathrm{supratentorial}\mathrm{volume})+\mathrm{covariates},$$

[4] $$\log_{10}(CT)∼\alpha^{*}\log_{10}(\mathrm{supratentorial}\mathrm{volume})+\mathrm{covariates}.$$

Supratentorial volume was used as the volumetric term because total SA and CT exclude infratentorial structures. The universal scaling model governing cortical folding is described in Eq. **5**, where α=1.25. By applying a logarithmic transformation and linear regressions, α can be calculated Eq. **6**.

[5] $$\mathrm{total}{\mathrm{SA}}^{*}{\mathrm{CT}}^{0.5}∼K^{*}\mathrm{exposed}{\mathrm{SA}}^{1.25},$$

[6] $$\log_{10}(\mathrm{total}{\mathrm{SA}}^{*}{\mathrm{CT}}^{0.5})∼\alpha^{*}\log_{10}(\mathrm{exposed}\mathrm{SA})+\mathrm{covariates}.$$

Scaling coefficients with 95% CI were calculated from the regression coefficient and SE for log(supratentorial volume) or log(exposed SA). The GVLMA package (https://cran.r-project.org/web/packages/gvlma) was used to check model fit and if models did not meet regression assumptions, robust regression with fast-S algorithms were used to estimate regression coefficients (50).

### Multivariate Morphological Terms.

Geometrically, each cortex can be represented by a point across three axes [total SA, exposed SA, CT^2^] (*SI Appendix*, Fig. S1) (16) and the scaling law in Eq. **6** (without covariates) represents the plane on which cortices are expected to plot. Wang and colleagues (16) demonstrated that the deviation from this plane can be captured by isolating k, from Eq. **5**. By allowing k to be a free parameter and holding the scaling coefficients constant, a term capturing the offset, or deviance, from the plane described in Eq. **6** is calculated. K increases with higher total SA and CT but lower exposed SA, as would be expected for structures that are more folded:

[7] $$K=\log(K)=1^{*}\log(\mathrm{total}\mathrm{SA})-1.25^{*}\log(\mathrm{exposed}\mathrm{SA})+0.25^{*}\log({\mathrm{CT}}^{2}).$$

An isometric term, capturing increases in cortical size, can be calculated by multiplying all morphological variables by a common numerical factor (e.g. 1):

[8] $$I=\log(\mathrm{total}\mathrm{SA})+\log(\mathrm{exposed}\mathrm{SA})+\log({\mathrm{CT}}^{2}).$$

A final variable, the cross product of coefficients from offset [1, −1.25,0.25] and isometric terms [1,1,1], which Wang and colleagues (16) term “shape,” captures information about shape, independent of size or the offset Eq. **9**). It increases with higher total and exposed SA but lower CT:

[9] $$S=1.5^{*}\log(\mathrm{total}\mathrm{SA}+0.75^{*}\log(\mathrm{exposed}\mathrm{SA})-2.25^{*}\log({\mathrm{CT}}^{2}).$$

### Statistical Comparisons.

The linearHypothesis function in the CAR package (https://r-forge.r-project.org/projects/car/) was used to test whether observed regression coefficients in the typically developing controls were significantly different than the theoretically predicted values and observed coefficients in adults (9).

Multiple linear regressions with permutation testing (n = 5,000) were used for all further analyses. The effect of demographics (GA, PMA, PMA^2^, sex, birth-weight z-score, and multiple birth) on scaling coefficients and multivariate morphological terms in each group was assessed. To assess the effect on scaling coefficients, the interaction between each variable and log(supratentorial volume) or log(texposed SA) was assessed, adjusting for other demographic variables. For multivariate morphological terms, all variables were entered into the models as independent predictors adjusting for other demographic variables. The effect of days of parenteral nutrition and respiratory support in preterm infants, and CHD subgroup and cerebral oxygen delivery in infants with CHD were also assessed.

To assess the effect of group on scaling coefficients, the interaction between group and log(supratentorial volume) or log(exposed SA) was assessed. For multivariate morphological terms, group was entered into models as an independent predictor. Analyses were undertaken separately to assess the effect of group in preterm infants and controls, and preterm infants and infants with CHD adjusting for PMA, PMA^2^, sex, and multiple birth. To assess the effect of group in infants with CHD and controls, typically developing infants who underwent MRI outside of the PMA range of infants with CHD (37.14 to 42.29 wk) were removed (n = 112). The effect of group (control vs CHD) was assessed adjusting for PMA, PMA^2^, GA, sex, and multiple birth.

In order to characterize the association between scaling relationships, multivariate morphological terms, and neurodevelopmental outcomes in early childhood, an individual’s degree of deviation from the typical population across each metric was calculated as previously reported by Williams et al. (9). A complete robust regression model for each metric was constructed for the typically developing control population and then applied to infants with CHD and those born preterm. For each metric, residuals were extracted for all infants, and z-scored to the control residuals to obtain a z‐score measuring an individual’s deviance from the typical population, given their PMA, PMA^2^, sex, multiple birth status, birth weight z-score, and additionally log(supratentorial volume) or log(exposed SA) for scaling relationships. We assessed whether deviance z-scores predicted cognitive, language, and motor scores in early childhood in typically developing controls and whether there was an interaction between group and deviance z-scores adjusting for socioeconomic status. We also covaried for a parent speaking English as a second language in the language models. Bootstrapped R^2^ were calculated for each deviance z-score which predicted neurodevelopmental outcome scores.

Finally, we assessed whether group predicted individual cortical metrics (supratentorial brain volume, total SA, GI, and CT) and whether individual cortical metrics predicted outcome scores in each group, adjusting for PMA, PMA^2^, sex, multiple birth, birth weight z-score, socioeconomic status, and GA (for CHD v control models).

Holm family-wise error rate correction was applied to adjust for multiple comparisons (reported as pFWE).

## Supplementary Material

Appendix 01 (PDF)

Dataset S01 (CSV)

Code S01 (R)

## Data Availability

Developing human connectome project MRI and neurodevelopmental outcome data included in this study (controls and preterm infants, n = 418) are from the 3rd neonatal data release (18, 51). Anonymized derived data from 41 infants with CHD acquired under ethics 21/WA/0075 and infants from dHCP (n = 418), as well as an analysis script, are included as *SI Appendix*. We do not have ethical permission for sharing data for 66 infants with CHD acquired under ethics 07/H0707/105. Index of multiple deprivation rank is considered identifiable data and is therefore not included in the anonymized database.

## References

[1] R. Haartsen, E. J. Jones, M. H. Johnson, Human brain development over the early years. Curr. Opin. Behav. Sci. **10**, 149–154 (2016).

[2] I. Kostović, G. Sedmak, M. Judaš, Neural histology and neurogenesis of the human fetal and infant brain. Neuroimage **188**, 743–773 (2019). 30594683 10.1016/j.neuroimage.2018.12.043

[3] A. E. Lyall , Dynamic development of regional CTand SAin early childhood. Cereb. Cortex **25**, 2204–2212 (2015). 24591525 10.1093/cercor/bhu027 PMC4506327

[4] R. A. I. Bethlehem , Brain charts for the human lifespan. Nature **604**, 525–533 (2022). 35388223 10.1038/s41586-022-04554-y PMC9021021

[5] K. L. Grasby , The genetic architecture of the human cerebral cortex. Science **367**, eaay6690 (2020). 32193296 10.1126/science.aay6690 PMC7295264

[6] R. Dimitrova , Preterm birth alters the development of cortical microstructure and morphology at term-equivalent age. Neuroimage **243**, 118488 (2021). 34419595 10.1016/j.neuroimage.2021.118488 PMC8526870

[7] R. Rathbone , Perinatal cortical growth and childhood neurocognitive abilities. Neurology **77**, 1510–1517 (2011). 21998316 10.1212/WNL.0b013e318233b215 PMC3198973

[8] P. S. W. Boedhoe , Subcortical brain volume, regional cortical thickness, and cortical SA across disorders: Findings from the ENIGMA ADHD, ASD, and OCD working groups. Am. J. Psychiatry **177**, 834–843 (2020). 32539527 10.1176/appi.ajp.2020.19030331 PMC8296070

[9] C. M. Williams, H. Peyre, R. Toro, F. Ramus, Neuroanatomical norms in the UK Biobank: The impact of allometric scaling, sex, and age. Hum. Brain Mapp. **42**, 4623–4642 (2021). 34268815 10.1002/hbm.25572 PMC8410561

[10] P. K. Reardon , Normative brain size variation and brain shape diversity in humans. Science **360**, 1222–1227 (2018). 29853553 10.1126/science.aar2578 PMC7485526

[11] B. Mota, S. Herculano-Houzel, Cortical folding scales universally with surface area and thickness, not number of neurons. Science **349**, 74–77 (2015). 26138976 10.1126/science.aaa9101

[12] N. Demirci, M. A. Holland, Scaling patterns of cortical folding and thickness in early human brain development in comparison with primates. Cereb Cortex **34**, bhad462 (2024). 38271274 10.1093/cercor/bhad462

[13] O. Kapellou , Abnormal cortical development after premature birth shown by altered allometric scaling of brain growth. PLoS Med. **3**, e265 (2006). 16866579 10.1371/journal.pmed.0030265 PMC1523379

[14] R. A. Paul , An allometric scaling relationship in the brain of preterm infants. Ann. Clin. Transl. Neurol. **1**, 933–937 (2014). 25540808 10.1002/acn3.130 PMC4265065

[15] Y. Wang, J. Necus, M. Kaiser, B. Mota, Universality in human cortical folding in health and disease. Proc. Natl. Acad. Sci. U.S.A. **113**, 12820–12825 (2016). 27791126 10.1073/pnas.1610175113 PMC5111660

[16] Y. Wang , Independent components of human brain morphology. Neuroimage **226**, 117546 (2021). 33186714 10.1016/j.neuroimage.2020.117546 PMC7836233

[17] A. D. Edwards , The developing human connectome project neonatal data release. Front. Neurosci. **16**, 886772 (2022). 35677357 10.3389/fnins.2022.886772 PMC9169090

[18] C. J. Kelly , Impaired development of the cerebral cortex in infants with congenital heart disease is correlated to reduced cerebral oxygen delivery. Sci. Rep. **7**, 15088 (2017). 29118365 10.1038/s41598-017-14939-z PMC5678433

[19] A. Makropoulos , The developing human connectome project: A minimal processing pipeline for neonatal cortical surface reconstruction. Neuroimage **173**, 88–112 (2018). 29409960 10.1101/125526 PMC6783314

[20] N. Bayley, Bayley Scales of Infant and Toddler Development (PsychCorp/Pearson, ed. 3, 2006).

[21] C. Llinares-Benadero, V. Borrell, Deconstructing cortical folding: Genetic, cellular and mechanical determinants. Nat. Rev. Neurosci. **20**, 161–176 (2019). 30610227 10.1038/s41583-018-0112-2

[22] K. Im , Brain size and cortical structure in the adult human brain. Cereb. Cortex **18**, 2181–2191 (2008). 18234686 10.1093/cercor/bhm244

[23] M. Dibble, J. Z. Ang, L. Mariga, E. J. Molloy, A. L. W. Bokde, Diffusion Tensor imaging in very preterm, moderate-late preterm and term-born neonates: A systematic review. J. Pediatr. **232**, 48–58.e3 (2021). 33453200 10.1016/j.jpeds.2021.01.008

[24] A. Melbourne , Preterm birth affects the developmental synergy between cortical folding and cortical connectivity observed on multimodal MRI. Neuroimage **89**, 23–34 (2014). 24315841 10.1016/j.neuroimage.2013.11.048

[25] P. Rakic, Less is more: Progenitor death and cortical size. Nat. Neurosci. **8**, 981–982 (2005). 16047024 10.1038/nn0805-981

[26] S. C. Jha , Environmental influences on infant CT and surface area. Cereb. Cortex **29**, 1139–1149 (2019). 29420697 10.1093/cercor/bhy020 PMC6373689

[27] J. J. Volpe, Primary neuronal dysmaturation in preterm brain: Important and likely modifiable. J. Neonatal Perinatal Med. **14**, 1–6 (2021). 33136070 10.3233/NPM-200606 PMC7990400

[28] B. Schmitz-Koep , Aberrant allometric scaling of cortical folding in preterm-born adults. Brain Commun. **5**, fcac341 (2022). 36632185 10.1093/braincomms/fcac341 PMC9830984

[29] M. Barresi , Toward a better understanding of how a gyrified brain develops. Cereb. Cortex **34**, bhae055 (2024). 38425213 10.1093/cercor/bhae055

[30] A. M. Pagnozzi , Early brain morphometrics from neonatal MRI predict motor and cognitive outcomes at 2-years corrected age in very preterm infants. Neuroimage **267**, 119815 (2023). 36529204 10.1016/j.neuroimage.2022.119815

[31] K. J. Kersbergen , Relation between clinical risk factors, early cortical changes, and neurodevelopmental outcome in preterm infants. Neuroimage **142**, 301–310 (2016). 27395393 10.1016/j.neuroimage.2016.07.010

[32] J. E. Kline , Early cortical maturation predicts neurodevelopment in very preterm infants. Arch. Dis. Child Fetal Neonatal Ed. **105**, 460–465 (2020). 31704737 10.1136/archdischild-2019-317466 PMC7205568

[33] J. Lefèvre , Are developmental trajectories of cortical folding comparable between cross-sectional datasets of fetuses and preterm newborns?. Cereb. Cortex **26**, 3023–3035 (2016). 26045567 10.1093/cercor/bhv123

[34] L. Sun , Reduced fetal cerebral oxygen consumption is associated with smaller brain size in fetuses with congenital heart disease. Circulation **131**, 1313–1323 (2015). 25762062 10.1161/CIRCULATIONAHA.114.013051 PMC4398654

[35] P. D. Morton , Abnormal neurogenesis and cortical growth in congenital heart disease. Sci. Transl. Med. **9**, eaah7029 (2017). 28123074 10.1126/scitranslmed.aah7029 PMC5467873

[36] F.-T. Lee, M. Seed, L. Sun, D. Marini, Fetal brain issues in congenital heart disease. Transl. Pediatr. **10**, 2182–2196 (2020). 10.21037/tp-20-224 PMC8429876 34584890

[37] M. Bouyssi-Kobar , Delayed cortical development in fetuses with complex congenital heart disease. Cereb. Cortex **23**, 2932–2943 (2012). 22977063 10.1093/cercor/bhs281

[38] C. Limperopoulos , Brain volume and metabolism in fetuses with congenital heart disease: Evaluation with quantitative magnetic resonance imaging and spectroscopy. Circulation **121**, 26–33 (2010). 20026783 10.1161/CIRCULATIONAHA.109.865568 PMC2819908

[39] A. F. Bonthrone , Individualized brain development and cognitive outcome in infants with congenital heart disease. Brain Commun. **3**, fcab046 (2021). 33860226 10.1093/braincomms/fcab046 PMC8032964

[40] D. Cromb , Total and regional brain volumes in fetuses with congenital heart disease. J. Magn. Reson. Imaging **60**, 497–509 (2023). 37846811 10.1002/jmri.29078 PMC7616254

[41] C. Ortinau , Cortical folding is altered before surgery in infants with congenital heart disease. J. Pediatr. **163**, 1507–1510 (2013). 23988135 10.1016/j.jpeds.2013.06.045 PMC3905308

[42] N. H. P. Claessens , Delayed cortical gray matter development in neonates with severe congenital heart disease. Pediatr. Res. **80**, 668 (2016). 27434120 10.1038/pr.2016.145

[43] D. Cromb , Individualized cortical gyrification in neonates with congenital heart disease. Brain Commun. **65**, fcae356 (2024). 10.1093/braincomms/fcae356 PMC11487749 39429246

[44] C. J. Kelly , Abnormal microstructural development of the cerebral cortex in neonates with congenital heart disease is associated with impaired cerebral oxygen delivery. J. Am. Heart Assoc. **8**, e009893 (2019). 30821171 10.1161/JAHA.118.009893 PMC6474935

[45] M. Eyre , The Developing Human Connectome Project: Typical and disrupted perinatal functional connectivity. Brain **144**, 2199–2213 (2021). 33734321 10.1093/brain/awab118 PMC8370420

[46] A. K. Ewer , Pulse oximetry screening for congenital heart defects in newborn infants (PulseOx): A test accuracy study. Lancet **378**, 785–794 (2011). 21820732 10.1016/S0140-6736(11)60753-8

[47] E. J. Hughes , A dedicated neonatal brain imaging system. Magn. Reson. Med. **78**, 794–804 (2017). 27643791 10.1002/mrm.26462 PMC5516134

[48] L. Cordero-Grande, E. J. Hughes, J. Hutter, A. N. Price, J. V. Hajnal, Three-dimensional motion corrected sensitivity encoding reconstruction for multi-shot multi-slice MRI: Application to neonatal brain imaging. Magn. Reson. Med. **79**, 1365–1376 (2018). 28626962 10.1002/mrm.26796 PMC5811842

[49] A. Makropoulos , Automatic whole brain MRI segmentation of the developing neonatal brain. IEEE Trans. Med. Imaging **33**, 1818–1831 (2014). 24816548 10.1109/TMI.2014.2322280

[50] M. Maechler , robustbase: Basic Robust Statistics (Version 0.99.2, 2024). https://robustbase.r-forge.r-project.org/. Accessed 2 February 2024.

[51] A. D. Edwards, J. V. Hajnal, D. Rueckert, S. Smith, Developing Human Connectome Project (dHCP). National Institute for Mental Health (NIMH) Dara Archive. https://nda.nih.gov/edit_collection.html?id=3955. Deposited 1 November 2021.

