# Supplementary material for: Cortical scaling of the neonatal brain in typical and altered development
Source: Proc Natl Acad Sci U S A. 2025 Apr 8;122(15):e2416423122. doi: 10.1073/pnas.2416423122 (PMC12012530; doi:10.1073/pnas.2416423122)
Supplement: Supplementary file 1 — Appendix 01 (PDF) [file pnas.2416423122.sapp.pdf]

Cortical scaling of the neonatal brain in typical and altered development

Alexandra F. Bonthrone[1], Daniel Cromb[1], Andrew Chew[1], Barat Gal-Er[1], Christopher Kelly[1], Shona Falconer[1], Tomoki Arichi[1,2,3], Kuberan Pushparajah[4,5], John Simpson[5], Mary A. Rutherford[1,3], Joseph V. Hajnal[1], Chiara Nosarti[1,6], A. David Edwards[1,3], Jonathan O'Muircheartaigh[1,3,7], Serena J. Counsell[1]*

1 Centre for the Developing Brain, Research Department of Early Life Imaging, School of Biomedical Engineering and Imaging Sciences, King's College London, London, SE1 7EH, UK

2 Paediatric Neurosciences, Evelina London Children's Hospital, London

3 MRC Centre for Neurodevelopmental Disorders, King's College London

4 Department of Cardiovascular Imaging, King's College London, London, UK

5 Department of Fetal and Paediatric Cardiology, Evelina London Children's Hospital, London, UK

6 Department of Child and Adolescent Psychiatry, Institute of Psychiatry, Psychology and Neuroscience, King's College London, UK

7 Department of Forensic and Neurodevelopmental Sciences, Institute of Psychiatry, Psychology and Neuroscience, King's College London, UK

*Corresponding author: Prof. Serena J. Counsell, Centre for the Developing Brain, School of Biomedical Engineering and Imaging Sciences, King's College London, London SE1 7EH, UK. Phone: 02071887188. Email: Serena.Counsell@kcl.ac.uk

This PDF file includes:

Tables S1 to S9
Figures S1 to S7
Supplementary Methods

Table S1. Sample characteristics				
	Controls (n=345)	Preterms (n=73)	CHD (n=107)	Group differences
Gestational age at birth, median (IQR)	40.14 (39.00-40.86)	29.14 (27.43-31.29)	38.57 (38.07-38.93)	$H(76)=224$ $p<0.001$[a]
Postmenstrual age at Scan, median (IQR)	41.43 (40.29-42.86)	41.29 (40.00-42.86)	39.00 (38.57-39.71)	$H(53)=164$ $p<0.001$[a]
Male, n (%)	179 (51.9)	39 (53.4)	60 (56.1)	$X^2 = 0.583$ $p=0.747$[b]
Index of Multiple Deprivation Quintile				
1 (most deprived)	52 (15.1)	13 (17.8)	17 (15.9)	$X^2 = 26.63$ $p<0.001$[b]
2	138 (40.0)	23 (31.5)	18 (16.8)	
3	70 (20.3)	19 (26.0)	26 (23.4)	
4	32 (9.3)	4 (5.5)	19 (17.8)	
5	51 (14.8)	14 (19.2)	26 (24.3)	
Not Available	2 (0.5)	0 (0)	2 (1.9)	
Primary Cardiac Diagnosis				
Abnormal mixing				
Transposition of the great arteries (TGA)	-	-	45	
Truncus arteriosus	-	-	3	
Double outlet right ventricle	-	-	3	
Total anomalous pulmonary venous drainage	-	-	1	
Left sided heart lesions				
Aortic arch anomalies[c]	-	-	25	
Hypoplastic left heart syndrome	-	-	7	
Aortic Stenosis	-	-	3	
Right sided heart lesions				
Tetralogy of Fallot	-	-	13	
Pulmonary atresia	-	-	4	
Pulmonary stenosis	-	-	2	
Tricuspid atresia	-	-	1	
Clinical variables				
days of respiratory support, median (IQR)	-	11 (2-44)	0 (0-5)[f]	
days of parenteral nutrition, median (IQR)	-	11 (5-16)	-	
Cerebral oxygen delivery (mLO_2/min), median (IQR)	-	-	1625 (1440-2088)	
Antenatal CHD diagnosis, n (%)	-	-	100 (93)	
Birth away from cardiac center, n (%)	-	-	10 (9)	
Neurodevelopmental follow-up assessments				
Number with follow-up	345 (100)	57 (78)	65 of 88	$X^2 = 0.012$ $p=0.914$[b]

assessment, n (%)			infants eligible for follow-up (74)[d]	(excluding controls)
Corrected age at assessment, median (IQR)	18.4 (18.1-19.2)	18.6 (18.2-19.3)	22.29 (22.06-23.84)	H(168)=374 p<0.001[a]
Cognitive Composite Score, median (IQR)	100 (95-110)	100 (90-105)	95 (85-100)	H(13)=46.6 p<0.001[a]
Motor Composite Score, median (IQR)	103 (97-110)	97 (91-107)	94 (91-103)	H(21)=59.1 p<0.001[a]
Language Composite Score, median (IQR)	100 (89-109)	100 (86-112)	91 (79-103)	H(29)=39.8 p=0.086[a]
Parents first language not English, n (%)[e]	152 (44.1)	15 (26.3)	15 (25)	X^2 = 13.6 p=0.001[b]

[a]Kruskal wallis test [b]X^2 test; [c]Coarctation of the aorta n=22, hypoplastic aortic arch n=2 or interrupted aortic arch n=1; [d]19 infants too young for follow-up; [e]Missing data controls n=14; preterms n=3; CHD n=5; [f]effect on allometric scaling not assessed

**Table S2**. Associations between demographic variables, allometric scaling and multivariate morphological features in typically developing infants			
**Total Surface Area Scaling**			
Model	B (95% CI)		p (pFWE)
Gestational age at birth	0.020 (-0.014- 0.054)	1.17	0.239 (0.957)
Postmenstrual age at scan	-0.014 (-0.042- 0.014)	-0.965	0.350 (1.00)
Postmenstrual age at scan^2	<-0.001 (<-0.001- 0.001)	-0.974	0.336 (1.00)
Male sex	-0.078 (-0.144- -0.011)	-2.31	0.024 (0.145)
Multiple birth	-0.067 (-0.243- 0.108)	-0.754	0.451 (1.00)
Birth weight z-score	-0.032 (-0.064- 0.001)	-1.94	0.051 (0.255)
**Cortical Thickness Scaling**			
Gestational age at birth	-0.025 (-0.057- 0.006)	-1.58	0.113 (0.453)
Postmenstrual age at scan	-0.014 (-0.040- 0.013)	-1.02	0.307 (0.921)
Postmenstrual age at scan^2	<-0.001 (<-0.001- 0.001)	-0.970	0.338 (0.921)
Male sex	0.062 (<-0.001- 0.124)	1.96	0.052 (0.259)
Multiple birth	0.180 (0.017- 0.344)	2.17	0.029 (0.173)
Birth weight z-score	-0.010 (-0.04- 0.020)	-0.641	0.538 (0.921)
**Cortical Folding Scaling**			
Gestational age at birth	0.021 (-0.024- 0.066)	0.931	0.365 (1.00)
Postmenstrual age at scan	0.036 (-0.003- 0.074)	1.84	0.062 (0.270)
Postmenstrual age at scan^2	<0.001 (<-0.001- <0.001)	1.94	0.054 (0.270)
Male sex	0.014 (-0.075- 0.103)	0.305	0.756 (1.00)
Multiple birth	-0.046 (-0.271- 0.180)	-0.400	0.690 (1.00)
Birth weight z-score	-0.107 (-0.149- -0.066)<br>*-0.003 (-0.034- 0.028)*	-5.11<br>*0.165*	<0.001 (<0.001)*<br>*0.869*[c]
**Multivariate offset term**[b]			
Gestational age at birth	-0.003 (-0.005- <-0.001)	-2.22	0.029 (0.145)
Postmenstrual age at scan	<0.001 (-0.062- 0.063)	0.987	0.987 (1.00)
Postmenstrual age at scan^2	<0.001 (-0.001- 0.001)	0.541	0.593 (1.00)
Male sex	-0.001 (-0.006- 0.003)	-0.633	0.519 (1.00)
Multiple birth	-0.011 (-0.024- 0.002)	-1.60	0.107 (0.428)
Birth weight z-score	0.004 (0.001-0.006)	3.02	0.004 (0.024)*
**Multivariate isometric term**[b]			
Gestational age at birth	-0.030 (-0.041- -0.019)	-5.30	<0.001 (<0.001)*
Postmenstrual age at scan	-0.208 (-0.501- 0.085)	-1.40	0.159 (0.210)
Postmenstrual age at scan^2	0.004 (<-0.001- 0.007)	2.01	0.05 (0.150)
Male sex	0.027 (0.007- 0.047)	2.63	0.01 (0.040)*
Multiple birth	-0.05 (-0.111- 0.011)	-1.60	0.105 (0.210)
Birth weight z-score	0.043 (0.032-0.055)	7.64	<0.001 (<0.001)*
**Multivariate shape term**[b]			
Gestational age at birth	0.040 (0.019- 0.061)	3.78	<0.001 (0.001)*
Postmenstrual age at scan	0.541 (-0.006- 1.09)	1.95	0.052 (0.208)
Postmenstrual age at scan^2	-0.007 (-0.013- <0.001)	-2.05	0.039 (0.195)
Male sex	0.019 (-0.019- 0.057)	1.01	0.305 (0.915)
Multiple birth	0.046 (-0.069- 0.161)	0.790	0.424 (0.915)
Birth weight z-score	-0.003 (-0.024- 0.018)	-0.287	0.765 (0.915)
*significant p_{FWE}<0.05; [a]Interaction between demographic variable and log(supratentorial			

volume) or log(exposed SA) adjusting for other demographic variables; [b] independent predictor adjusting for other demographic variables; [c]*P*resence of outliers noted on visual inspection. Models run with robust regression, and the relationship was longer significant

Table S3. Associations between demographic variables, allometric scaling and multivariate morphological features in preterm infants

Model	B (95% CI)	t-score	P (p_{FWE})
**Total Surface Area Scaling**			
Model	B (95% CI)	t-score	P (p_{FWE})
Gestational age at birth	-0.007 (-0.029- 0.014)	-0.670	0.504 (1.00)
Postmenstrual age at scan	-0.015 (-0.052- 0.023)	-0.789	0.434 (1.00)
Postmenstrual age at scan^2	<-0.001 (-0.001- <0.001)	-0.805	0.424 (1.00)
Male sex	-0.056 (-0.161- 0.049)	-1.06	0.285 (1.00)
Multiple birth	0.067 (-0.100- 0.234)	0.804	0.417 (1.00)
Birth weight z-score	-0.011 (-0.063- 0.041)	-0.407	0.692 (1.00)
**Cortical Thickness Scaling**			
Gestational age at birth	-0.005 (-0.028- 0.018)	-0.403	0.675 (1.00)
Postmenstrual age at scan	0.010 (-0.030- 0.049)	0.491	0.612 (1.00)
Postmenstrual age at scan^2	<0.001 (<-0.001- 0.001)	0.525	0.601 (1.00)
Male sex	0.081 (-0.029- 0.192)	1.47	0.141 (0.705)
Multiple birth	0.003 (-0.175- 0.180)	0.030	0.975 (1.00)
Birth weight z-score	0.047 (-0.007- 0.101)	1.75	0.086 (0.516)
**Cortical Folding Scaling**			
Gestational age at birth	<-0.001 (<-0.001- <0.001)	-0.281	0.771 (1.00)
Postmenstrual age at scan	-0.007 (-0.054- 0.040)	-0.289	0.786 (1.00)
Postmenstrual age at scan^2	-0.010 (-0.074- 0.054)	-0.319	0.750 (1.00)
Male sex	-0.042 (-0.172- 0.088)	-0.643	0.534 (1.00)
Multiple birth	0.077 (-0.122- 0.277)	0.773	0.428 (1.00)
Birth weight z-score	0.004 (-0.023- 0.031)	0.308	0.743 (1.00)
**Multivariate offset term**[b]			
Gestational age at birth	0.003 (0.002-0.004)	4.27	<0.001 (<0.001)*
Postmenstrual age at scan	0.132 (0.039- 0.225)	2.83	0.006 (0.030)
Postmenstrual age at scan^2	-0.001 (-0.003- <-0.001)	-2.46	0.015 (0.060)
Male sex	0.001 (-0.006- 0.009)	0.344	0.731 (0.972)
Multiple birth	0.003 (-0.006- 0.013)	0.701	0.486 (0.972)
Birth weight z-score	0.003 (-0.001- 0.007)	1.68	0.096 (0.288)
**Multivariate isometric term**[b]			
Gestational age at birth	0.017 (0.009- 0.026)	4.11	<0.001 (0.001)*
Postmenstrual age at scan	1.05 (0.505- 1.60)	3.84	<0.001 (0.001)*
Postmenstrual age at scan^2	-0.02 (-0.018- -0.005)	-3.56	<0.001 (0.001)*
Male sex	0.026 (-0.018- 0.070)	1.18	0.238 (0.238)
Multiple birth	0.083 (0.025-1.41)	2.84	0.006 (0.012)
Birth weight z-score	0.047 (0.025-0.069)	4.29	<0.001 (<0.001)*
**Multivariate shape term**[b]			
Gestational age at birth	0.015 (<-0.001- 0.030)	1.95	0.055 (0.330)
Postmenstrual age at scan	0.008 (-0.978-0.994)	0.016	0.988 (1.00)
Postmenstrual age at scan^2	<0.001 (-0.012- 0.012)	-0.039	0.966 (1.00)
Male sex	0.076 (-0.003- 0.154)	1.91	0.063 (0.330)
Multiple birth	<0.001 (-0.105-0.105)	0.008	0.993 (1.00)
Birth weight z-score	0.020 (-0.020- 0.059)	0.999	0.312 (1.00)

* significant $p_{FWE}<0.05$; [a]Interaction between demographic variable and log(supratentorial volume) or log(exposed SA) adjusting for other demographic variables; [b] independent predictor

adjusting for other demographic variables;

**Table S4.** Associations between demographic variables, allometric scaling and multivariate morphological features in infants with CHD			
**Total Surface Area Scaling**			
Model	B (95% CI)	t-score	P (p_{FWE})
Gestational age at birth	-0.006 (-0.080- 0.067)	-0.173	0.850 (1.00)
Postmenstrual age at scan	-0.020 (-0.086- 0.046)	-0.599	0.552 (1.00)
Postmenstrual age at scan^2	<-0.001 (-0.001- 0.001)	-0.642	0.533 (1.00)
Male sex	-0.035 (-0.173- 0.103)	-0.508	0.592 (1.00)
Multiple birth	0.201 (-0.173- 0.575)	1.07	0.287 (1.00)
Birth weight z-score	-0.033 (-0.086- 0.020)	-1.24	0.224 (1.00)
**Cortical Thickness Scaling**			
Gestational age at birth	-0.034 (-0.109- 0.042)	-0.876	0.383 (1.00)
Postmenstrual age at scan	-0.025 (-0.094- 0.043)	-0.731	0.456 (1.00)
Postmenstrual age at scan^2	<-0.001 (-0.001- 0.001)	-0.705	0.472 (1.00)
Male sex	0.034 (-0.110- 0.177)	0.466	0.639 (1.00)
Multiple birth	-0.048 (-0.440- 0.343)	-0.244	0.805 (1.00)
Birth weight z-score	0.010 (-0.045- 0.065)	0.360	0.717 (1.00)
**Cortical Folding Scaling**			
Gestational age at birth	-0.003 (-0.109- 0.103)	-0.054	0.953 (1.00)
Postmenstrual age at scan	-0.015 (-0.106- 0.075)	-0.334	0.740 (1.00)
Postmenstrual age at scan^2	<-0.001 (-0.001- 0.001)	-0.318	0.751 (1.00)
Male sex	-0.009 (-0.179- 0.160)	-0.108	0.915 (1.00)
Multiple birth	0.231 (-0.237- 0.700)	0.970	0.332 (1.00)
Birth weight z-score	-0.030 (-0.097- 0.036)	-0.900	0.366 (1.00)
**Multivariate offset term**[b]			
Gestational age at birth	-0.001 (-0.009- 0.006)	-0.346	0.733 (1.00)
Postmenstrual age at scan	0.086 (-0.077-0.250)	1.04	0.297 (1.00)
Postmenstrual age at scan^2	-0.001 (-0.003- 0.001)	-0.831	0.411 (1.00)
Male sex	-0.003 (-0.009- 0.003)	-1.02	0.315 (1.00)
Multiple birth	-0.007 (-0.020- 0.005)	-1.14	0.263 (1.00)
Birth weight z-score	0.003 (<0.001-0.006)	1.97	0.054 (0.324)
**Multivariate isometric term**[b]			
Gestational age at birth	0.004 (-0.046- 0.054)	0.158	0.873 (1.00)
Postmenstrual age at scan	-0.350 (-1.426-0.726)	-0.645	0.514 (1.00)
Postmenstrual age at scan^2	0.005 (-0.008- 0.019)	0.756	0.461 (1.00)
Male sex	0.026 (-0.012- 0.063)	1.37	0.169 (0.845)
Multiple birth	-0.044 (-0.126- 0.038)	-1.06	0.281 (1.00)
Birth weight z-score	0.039 (0.019- 0.059)	3.83	<0.001 (0.002)*
**Multivariate shape term**[b]			
Gestational age at birth	-0.063 (-0.158- 0.032)	-1.32	0.183 (0.915)
Postmenstrual age at scan	1.37 (-0.679-3.43)	1.32	0.190 (0.915)
Postmenstrual age at scan^2	-0.016 (-0.042- 0.01)	-1.228	0.229 (0.915)
Male sex	0.101 (0.030- 0.173)	2.80	0.007 (0.042)
Multiple birth	0.051 (-0.105- 0.207)	0.645	0.511 (1.00)
Birth weight z-score	-0.011 (-0.049- 0.027)	-0.559	0.574 (1.00)
* significant $p_{FWE}<0.05$; [a]Interaction between demographic variable and log(supratentorial volume) or log(exposed SA) adjusting for other demographic variables; [b]independent predictor			

adjusting for other demographic variables.

**Table S5.** Associations between clinical variables, allometric scaling and multivariate morphological features in preterm infants and infants with CHD			
**Preterm infants**			
**Days of respiratory support**[a]			
Model	B (95% CI)	t-score	P (p_{FWE})
Total Surface Area Scaling[b]	0.001 (-0.001- 0.003)	0.853	0.382 (1.00)
Cortical Thickness Scaling[b]	0.001 (-0.001- 0.003)	1.15	0.253 (1.00)
Cortical Folding Scaling[b]	0.001 (-0.001- 0.003)	0.740	0.466 (1.00)
Multivariate offset term[c]	<0.001 (<-0.001- 0.001)	0.895	0.371 (1.00)
Multivariate isometric term[c]	<0.001 (-0.001- 0.001)	0.221	0.825 (1.00)
Multivariate shape term[c]	0.001 (-0.001- 0.003)	0.785	0.434 (1.00)
**Days of parenteral nutrition**			
Total Surface Area Scaling[b]	0.002 (-0.001- 0.004)	1.43	0.152 (1.00)
Cortical Thickness Scaling[b]	0.001 (-0.002- 0.004)	0.731	0.468 (1.00)
Cortical Folding Scaling[b]	0.002 (-0.001- 0.005)	1.21	0.226 (1.00)
Multivariate offset term[c]	<-0.001 (<-0.001- <0.001)	-0.833	0.397 (1.00)
Multivariate isometric term[c]	-0.001 (-0.002- <0.001)	-1.79	0.077 (0.919)
Multivariate shape term[c]	0.001 (-0.001- 0.003)	0.867	0.382 (1.00)
**Babies with CHD**			
**CHD subcategory (reference: abnormal streaming v left sided lesions)**			
Total Surface Area Scaling[b]	-0.017 (-0.153- 0.120)	-0.240	0.806 (1.00)
Cortical Thickness Scaling[b]	0.062 (-0.073- 0.199)	0.911	0.365 (1.00)
Cortical Folding Scaling[b]	-0.080 (-0.255- 0.095)	-0.906	0.359 (1.00)
Multivariate offset term[c]	0.005 (-0.001- 0.012)	1.58	0.105 (1.00)
Multivariate isometric term[c]	0.081 (0.041- 0.121)	4.03	<0.001 (<0.001)*
Multivariate shape term[c]	-0.098 (-0.178- -0.018)	-2.42	0.016 (0.250)
**CHD subtype (reference: abnormal streaming v right sided lesions)**			
Total Surface Area Scaling[b]	0.025 (-0.161- 0.212)	0.268	0.791 (1.00)
Cortical Thickness Scaling[b]	0.111 (-0.075- 0.297)	1.18	0.234 (1.00)
Cortical Folding Scaling[b]	0.039 (-0.201- 0.279)	0.324	0.744 (1.00)
Multivariate offset term[c]	0.002 (-0.006- 0.009)	0.442	0.664 (1.00)
Multivariate isometric term[c]	0.015 (-0.032- 0.061)	0.618	0.548 (1.00)
Multivariate shape term[c]	-0.055 (-0.149- 0.038)	-1.17	0.255 (1.00)
**Cerebral oxygen delivery**			
Total Surface Area Scaling[b]	<-0.001 (<-0.001- <0.001)	-0.367	0.710 (1.00)
Cortical Thickness Scaling[b]	<-0.001 (<-0.001- <0.001)	-0.946	0.344 (1.00)
Cortical Folding Scaling[b]	<0.001 (<0.001- <0.001)	0.009	0.993 (1.00)
Multivariate offset term[c]	<0.001 (<-0.001- <0.001)	1.79	0.071 (1.00)
Multivariate isometric term[c]	<0.001 (<0.001- <0.001)	3.54	0.001 (0.020)*
Multivariate shape term[c]	<-0.001 (<-0.001- <0.001)	-0.493	0.616 (1.00)
* significant $p_{FWE}<0.05$; [a]continuous positive airway pressure and mechanical ventilation; [a]Interaction between demographic variable and log(supratentorial volume) or log(exposed SA) adjusting for gestational age at birth, postmenstrual age at scan, postmenstrual age at scan^2, multiple birth, male sex and birth weight z-score; [b]independent predictor adjusting for gestational age at birth, postmenstrual age at scan, postmenstrual age at scan^2, multiple birth, male sex and birth weight z-score.			

**Table S6.** Associations between scaling relationship and multivariate morphological term deviance z-scores and cognitive language and motor abilities in typically developing infants			
Composite score	B (95% CI)	t-score	p
**Cortical folding scaling**			
cognition	0.199 (-0.923- 1.32)	0.349	0.720
language	-0.237 (-1.81- 1.34)	-0.296	0.763
motor	0.382 (-0.63- 1.40)	0.739	0.439
**multivariate isometric term**			
cognition	0.296 (-0.830- 1.42)	0.517	0.614
language	-0.256 (-1.85- 1.34)	-0.316	0.750
motor	-0.586 (-1.60- 0.432)	-1.13	0.258
**multivariate offset term**			
cognition	-0.028 (-1.15- 1.10)	-0.049	0.963
language	-0.436 (-2.01- 1.14)	-0.544	0.582
motor	-0.025 (-1.04- 0.992)	-0.049	0.963
t**otal surface area scaling**			
cognition	0.651 (-0.468- 1.77)	1.14	0.255
language	0.323 (-1.25- 1.90)	0.403	0.696
motor	0.510 (-0.504- 1.52)	0.990	0.321
**multivariate shape term**			
cognition	0.656 (-0.462- 1.78)	1.15	0.252
language	0.318 (-1.27- 1.90)	0.394	0.690
motor	0.590 (-0.423- 1.60)	1.15	0.249
**cortical thickness scaling**			
cognition	-0.383 (-1.50- 0.737)	-0.67	0.506
language	-0.320 (-1.90- 1.26)	-0.397	0.694
motor	-0.640 (-1.65- 0.373)	-1.24	0.210
adjusting for index of multiple deprivation (socioeconomic status). If a parent speaks English as a second language was included as an additional covariate in the language models.			

**Table S7.** Whole brain cortical metrics across all groups			
Metric	Controls	Preterm	CHD
Total surface area (cm^2)	974 (871-108)	900 (816- 105)	792 (721-845)
Cortical thickness (mm)	1.11 (1.07-1.14)	1.14 (1.10- 1.17)	1.08 (1.06-1.10)
Gyrification index (total surface area/exposed surface area)	2.62 (2.47-2.73)	2.44 (2.33- 2.60)	2.37 (2.27-2.46)
Supratentorial volume (cm^3)	335 (309-368)	325 (300- 364)	280 (262-298)
**Effect of group in typically developing controls (reference) and preterm infants**			
	B (95% CI)	t-score	p (p_{FWE})
Total surface area	-40.7 (-62.8- -18.5)	-3.61	<0.001 (0.002)*
Cortical thickness	0.028 (0.017- 0.040)	4.85	<0.001 (<0.001)*
Gyrification index	-0.138 (-0.170- -0.105)	-8.33	<0.001 (<0.001)*
Supratentorial volume	-7.23 (-14.7- -2.79)	-1.89	0.063 (0.190)
**Effect of group in typically developing controls (reference) and infants with CHD**			
Total surface area	-57.3 (-67.4- -27.1)	-4.62	<0.001 (<0.001)*
Cortical thickness	<-0.001 (-0.011- 0.010)	-0.063	0.949 (0.949)
Gyrification index	-0.043 (-0.074- -0.012)	-2.73	0.006 (0.015)*
Supratentorial volume	-22.1 (-28.9- -15.4)	-6.44	<0.001 (<0.001)*
**Effect of group in preterm infants (reference) and infants with CHD**			
Total Surface Area	-55.8 (-33.7- 22.5)	-0.392	0.701 (0.701)
Cortical thickness	-0.023 (-0.040- -0.007)	-2.80	0.005 (0.015)*
Gyrification index	0.095 (0.055-0.135)	4.68	<0.001 (<0.001)*
Supratentorial volume	-12.1 (-22.9- 13.1)	-2.21	0.031 (0.062)
*significant p_{FWE}<0.05; adjusting for Postmenstrual age at scan, Postmenstrual age at scan^2, Male sex, multiple birth and birth weight z-score. Gestational age at birth was an additional covariate for CHD vs controls.			

**Table S8.** Associations between cortical metrics and neurodevelopmental outcomes in typically developing controls, preterm infants and CHD						
Metric	B	2.5% CI bound	97.5% CI bound	-score	p	p_{FWE}
**Cognitive composite score**						
Typically developing controls						
Mean cortical thickness	-5.45	-32.93	22.03	-0.390	0.685	1.00
Supratentorial brain volume	<0.001	<0.001	<0.001	1.57	0.12	1.00
Total surface area	<0.001	<0.001	<0.001	1.79	0.078	0.86
Gyrification index	4.38	-4.74	13.50	0.94	0.348	1.00
Preterm infants						
Mean cortical thickness	-107.76	-183.73	-31.79	-2.85	0.006	0.067
Supratentorial brain volume	<0.001	<0.001	<0.001	-0.619	0.542	1.00
Total surface area	<0.001	<0.001	<0.001	-0.105	0.916	1.00
Gyrification index	26.72	-15.00	68.43	1.29	0.202	1.00
Infants with CHD						
Mean cortical thickness	36.77	-30.65	104.19	1.09	0.272	1.00
Supratentorial brain volume	<0.001	<0.001	<0.001	0.213	0.834	1.00
Total surface area	<0.001	<0.001	<0.001	0.392	0.705	1.00
Gyrification index	8.23	-19.98	36.45	0.585	0.568	1.00
**Language composite score**						
Typically developing controls						
Mean cortical thickness	-0.57	-38.90	37.75	-0.029	0.976	1.00
Supratentorial brain volume	<0.001	<0.001	<0.001	0.335	0.743	1.00
Total surface area	<0.001	<0.001	<0.001	0.308	0.747	1.00
Gyrification index	-1.37	-14.01	11.27	-0.214	0.832	1.00
Preterm infants						
Mean cortical thickness	-189.02	-286.96	-91.09	-3.89	0.001	0.012 *
Supratentorial brain volume	<0.001	<0.001	<0.001	-1.20	0.236	1.00
Total surface area	<0.001	<0.001	<0.001	-0.352	0.738	1.00
Gyrification index	50.62	-2.72	103.96	1.91	0.062	0.679
Infants with CHD						
Mean cortical thickness	45.23	-65.38	155.85	0.824	0.423	1.00
Supratentorial brain volume	<0.001	<0.001	<0.001	0.095	0.925	1.00
Total surface area	<0.001	<0.001	<0.001	0.825	0.413	1.00
Gyrification index	20.06	-23.56	63.68	0.927	0.369	1.00
**Motor composite scores**						
Typically developing controls						
Mean cortical thickness	-8.92	-33.93	16.09	-0.701	0.488	1.00
Supratentorial brain volume	<0.001	<0.001	<0.001	0.150	0.883	1.00
Total surface area	<0.001	<0.001	<0.001	0.400	0.680	1.00
Gyrification index	3.22	-5.09	11.53	0.761	0.437	1.00
Preterm infants						
Mean cortical thickness	-17.77	-90.56	55.03	-0.491	0.627	1.00
Supratentorial brain volume	<0.001	<0.001	<0.001	-1.40	0.172	1.00

Total surface area	<0.001	<0.001	<0.001	-1.09	0.276	1.00
Gyrification index	8.94	-28.61	46.49	0.479	0.642	1.00
Infants with CHD						
Mean cortical thickness	14.71	-49.57	79.00	0.459	0.642	1.00
Supratentorial brain volume	<0.001	<0.001	<0.001	-0.045	0.963	1.00
Total surface area	<0.001	<0.001	<0.001	0.082	0.938	1.00
Gyrification index	2.28	-24.55	29.11	0.171	0.869	1.00
adjusting for socioeconomic status, Male sex, Gestational age at birth, Postmenstrual age at scan, Postmenstrual age at scan^{2}, birth weight z-score and multiple birth.						

Table S9. Demographic characteristics of infants who attended follow-up compared to those who did not

	Preterm Infants			Infants with CHD		
	Attended follow-up (n=57)	Did not attend (n=16)		Attended follow-up (n=65)	Did not attend (n=23)	
Gestational age (weeks)	28.93 (27.43-31.00)	29.43 (24.57-31.14)	p=0.676[a]	38.71 38.29-39.14)	38.00 (37.45-38.50)	p=0.004[a]
Male	8 (47)	31 (55)	p=0.747[b]	34 (52)	16 (73)	p=0.154[b]
Index of multiple deprivation quintile	3 (2-3)	2 (1-3)	p=0.436[b]	3 (2-4)	3 (3-5)	p=0.172[b]
CHD subgroup	-	-	-	31 (48) abnormal mixing<br>23 (35) Left sided lesions	11 (50) abnormal mixing<br>7 (32) Left sided lesions	p>0.999[b]
Days of parenteral nutrition	11 (7-15)	11 (2-28)	p=0.965[a]	-	-	-
Days of respiratory support	11 (2-42)	14 (2-55)	p=0.789[a]	-	-	-

[a]Mann-witney U test; [b]X^2 test; variables presented as median (IQR) or n (%)

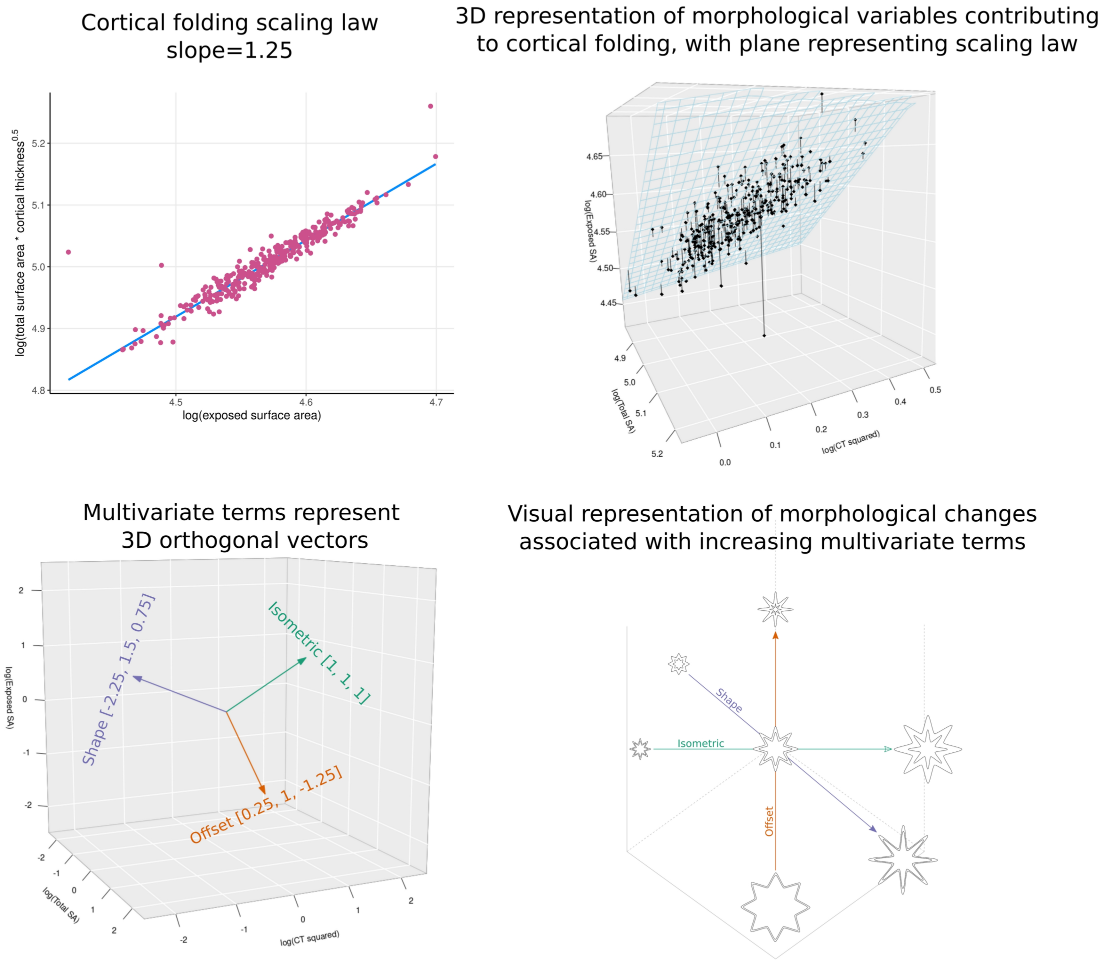

Figure S1. Illustration of the derivation of multivariate morphological terms from the cortical folding scaling law defined in Mota and Herculano-Hozel (11) based on Wang and Colleagues (16). The cortical folding scaling law defines a 3D plane capturing the relationship between Total SA, Exposed SA and CT^2 (16). The offset term represents the vector of orthogonal deviance from this 3D plane. The isometric term is a vector capturing increases in all cortical metrics. The Shape term is the vector product of the offset and isometric terms.

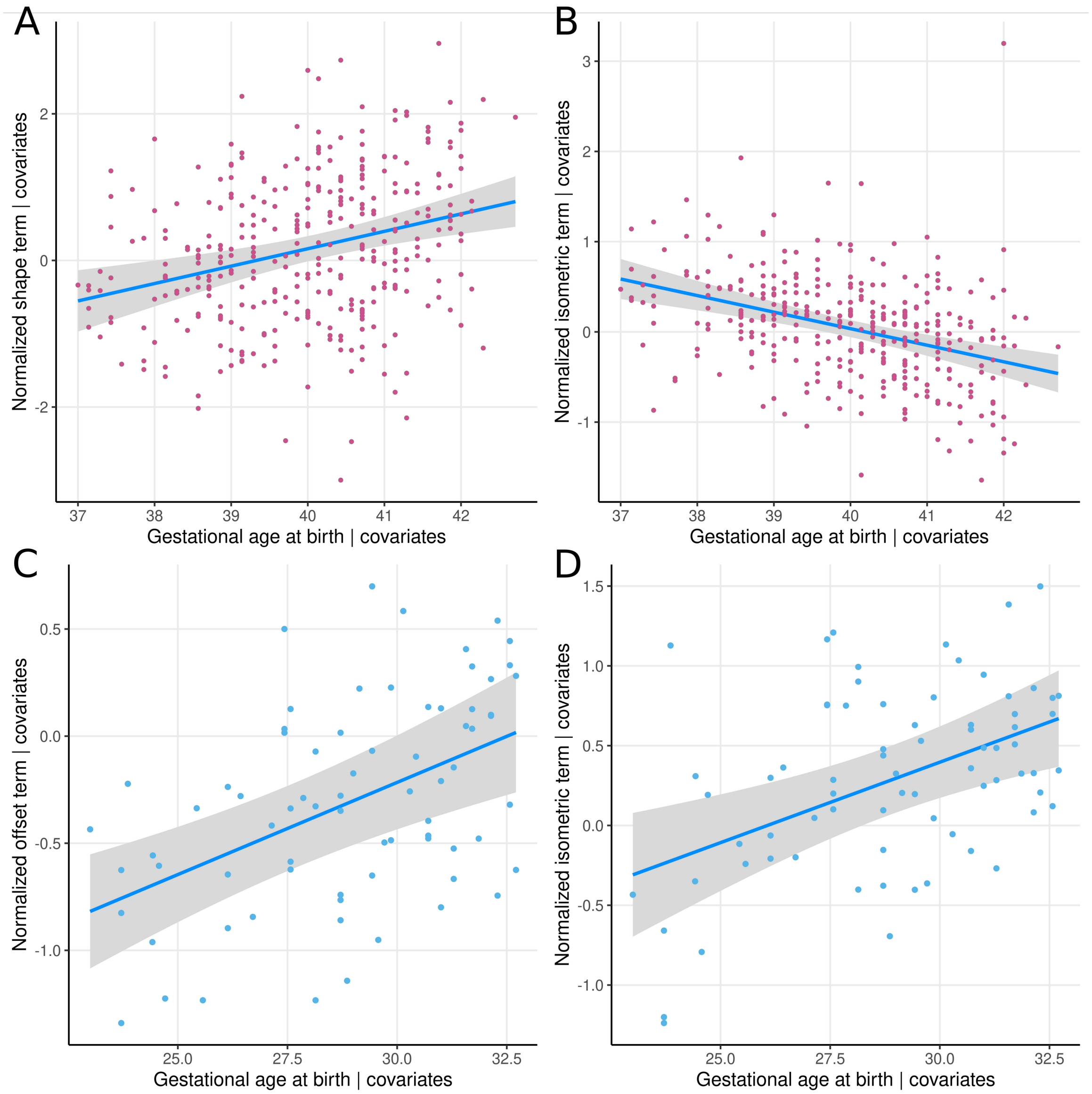

Figure S2. Associations between gestational age at birth and multivariate morphological terms (A,B) typically developing control infants and (C,D) preterm infants, adjusting for Postmenstrual age at scan, Postmenstrual age at scan^2 multiple birth, birth weight z-score and Male sex.

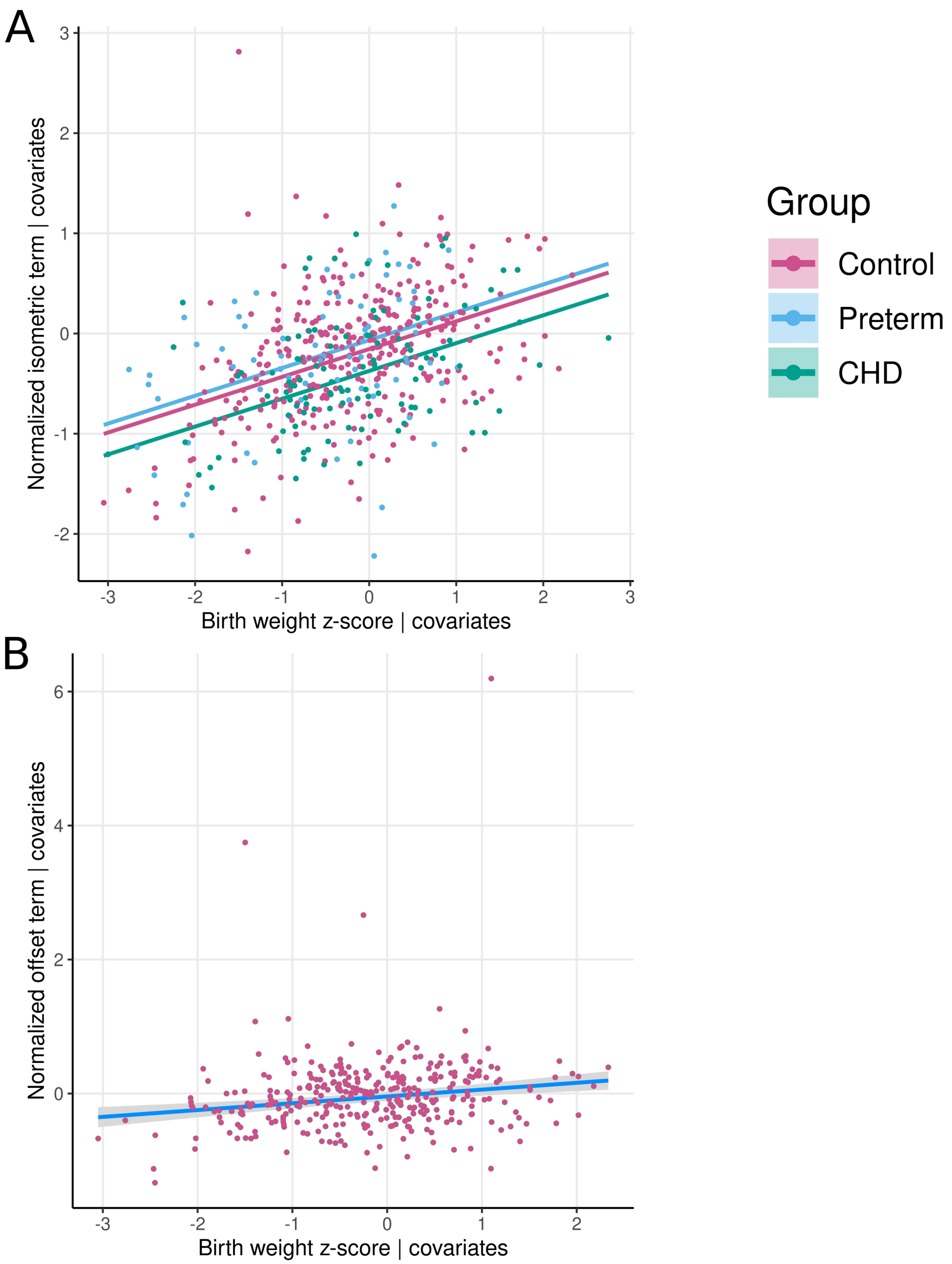

Figure S3. Associations between birth weight z-score and (A) isometric term across all groups and (B) offset term in typically developing control infants, adjusting for postmenstrual age at scan, postmenstrual age at scan^2 multiple birth and Male sex.

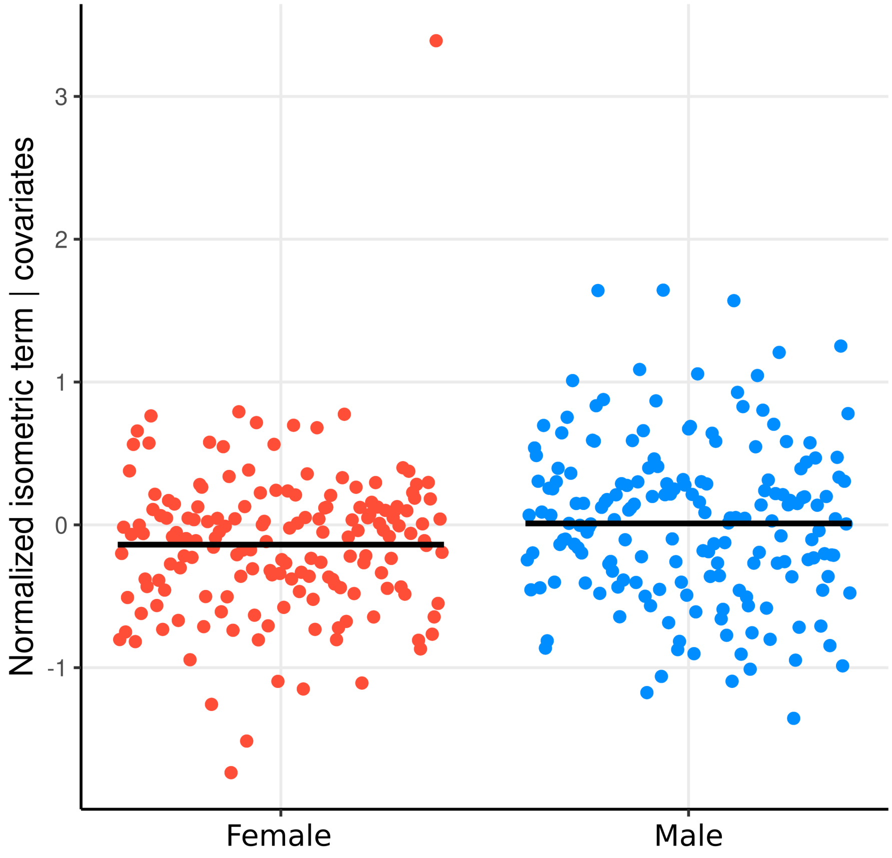

Figure S4. Association between isometric term and Male sex in typically developing infants, adjusting for postmenstrual age at scan, postmenstrual age at scan^2 multiple birth, birth weight z-score and gestational age at birth.

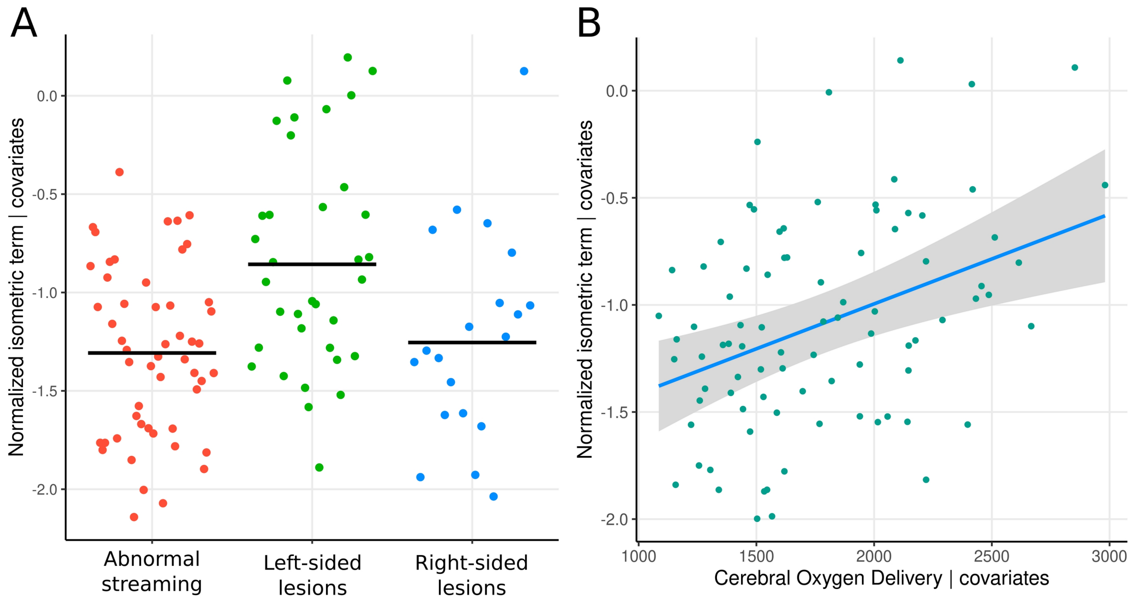

Figure S5. Associations between isometric term and (A) CHD subgroup (b) Cerebral oxygen delivery in infants with CHD adjusting for postmenstrual age at scan, postmenstrual age at scan^2, multiple birth, birth weight z-score, Male sex and gestational age at birth.

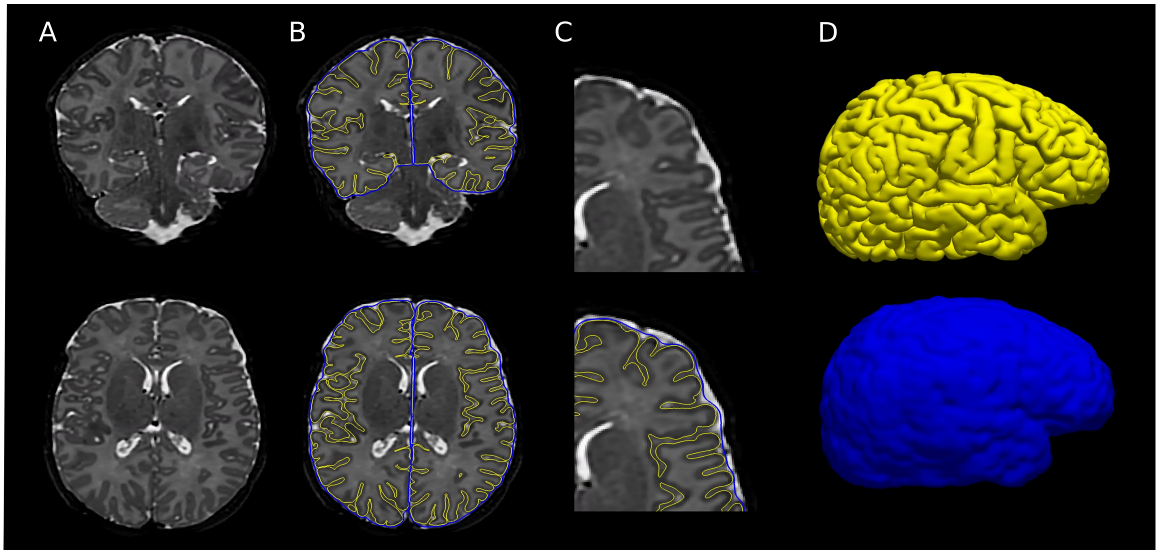

Figure S6. (A) T2-weighted MRI in a typically developing control infant. (B, C) Reconstruction of the pial surface (yellow) from which surface area is calculated, and exposed gyral surface (blue) from which outer surface area is calculated. (D) Pial and exposed gyral surface 3D reconstructions.

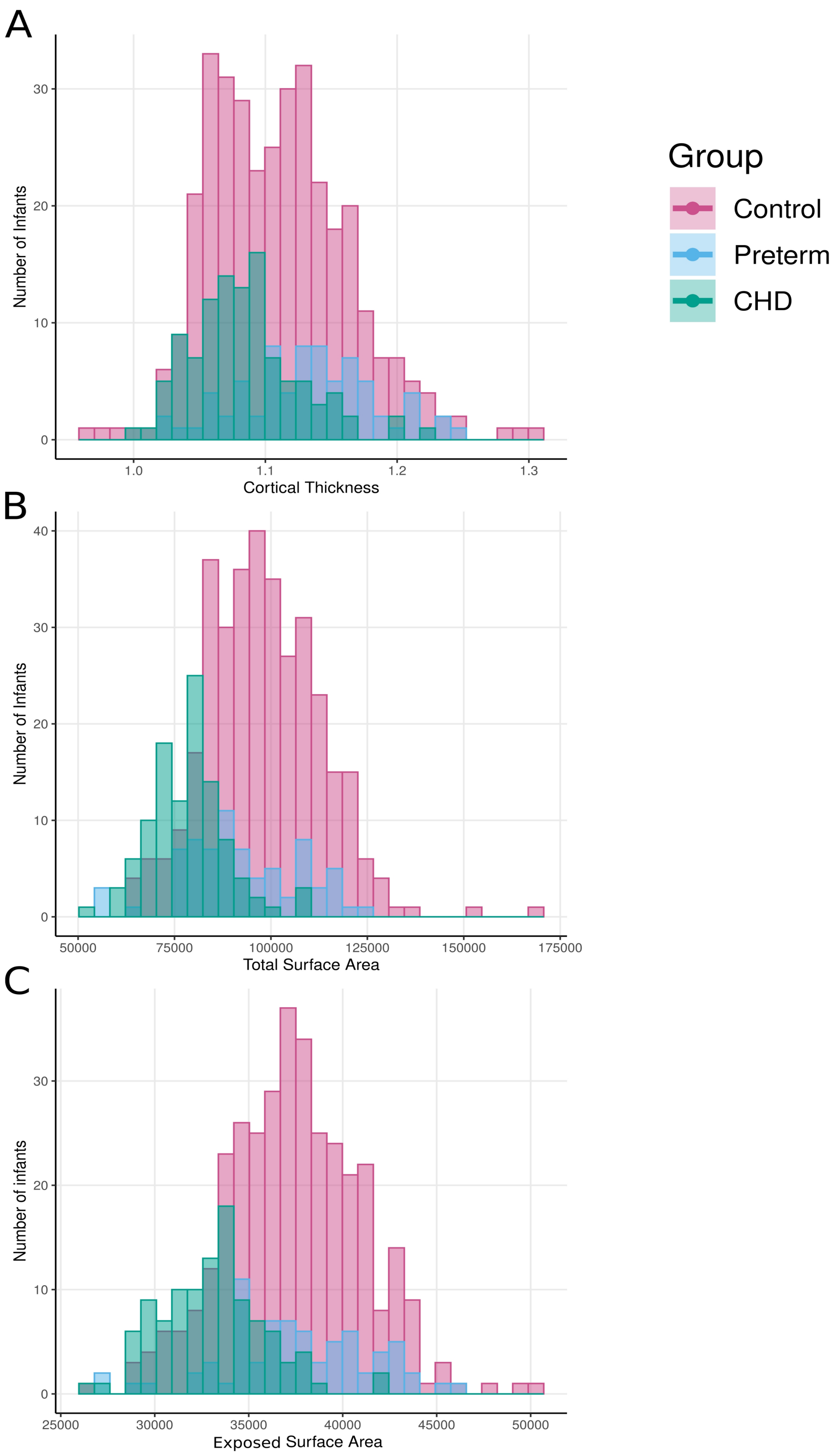

Figure S7. Histograms of (A) cortical thickness, (B), total surface area and (C) exposed surface area in each group.

Supplementary Methods

Quantitative flow imaging was performed in infants with CHD using velocity sensitized phase contrast imaging to measure cerebral blood flow. A single-slice T1-weighted fast-field-echo sequence was acquired in a plane perpendicular to both internal carotid and basilar arteries, at the level of the sphenoid bone using previously published methods (1) [100 × 100 mm^2, acquisition resolution 0.6 × 0.6 × 4.0 mm^2, TR 6.4 ms, TE 4.3 ms, flip angle 10°, 20 repetitions, maximal encoding velocity 140 cm/s, scan time: 71 s]. Regions of interest were drawn manually around the three vessels, using Segment v2.0 R480045 (2) and flow curves generated. An estimate of total cerebral blood flow was calculated from the sum of these vessels. Haemoglobin levels were measured as part of routine clinical care at a median of 2 days (range 0-9) prior to MRI. Pre-ductal arterial oxygen saturation was measured at the time of scan using a pulse-oximeter applied to the right hand. Cerebral oxygen delivery (CDO_2) was calculated using the following formula:

CDO2 (mLO_2/min) = Oxygen Saturation × Haemoglobin(g/dL) × 1.36 × Cerebral Blood Flow

(mL/min)

where 1.36 is the amount of oxygen bound per gram of haemoglobin at one atmosphere (Hüfner's constant).

References

1. C. J. Kelly, et al., Impaired development of the cerebral cortex in infants with congenital heart disease is correlated to reduced cerebral oxygen delivery. Sci Rep 7, 15088 (2017).

2. E. Heiberg, et al. "Design and validation of Segment-freely available software for cardiovascular image analysis." *BMC medical imaging* 10, 1-13 (2010).

Supplementary statistical methods

In table S1. differences in demographics data between groups was assessed with Kruskal wallis test for continuous data and X2 test for categorical variables. In table S2-4, the influence of demographic variables (GA, PMA, PMA2, sex, multiple birth and birth-weight z-score) on scaling relationships and multivariate morphological terms in typical infants (S2), preterm infants (S3) and infants with CHD (S4) using the following regression models:

Total surface area: log10(total SA) ~ demographic variable of interest*log10(supratentorial volume) + other demographic variables

Cortical thickness: log10(CT) ~ demographic variable of interest*log10(supratentorial volume) + other demographic variables

Cortical folding: log10(total SA*CT0.5) ~ demographic variable of interest*log10(exposed SA) + other demographic variables

Multivariate offset term: Offset term ~ demographic variable of interest + other demographic variables

Multivariate isometric term: Isometric term ~ demographic variable of interest + other demographic variables

Multivariate shape term: Shape term ~ demographic variable of interest + other demographic variables

In table S5, the impact of clinical variables (parenteral nutrition and mechanical ventilation in preterm infants, and CHD subtype and cerebral oxygen delivery in infants with CHD) on scaling relationships and multivariate morphological features in preterm infants and infants with CHD using the following regression models:

Total surface area: log10(total SA) ~ clinical variable of interest*log10(supratentorial volume) + demographic variables

Cortical thickness: log10(CT) ~ clinical variable of interest*log10(supratentorial volume) + demographic variables

Cortical folding: log10(total SA*CT0.5) ~ clinical variable of interest*log10(exposed SA) + demographic variables

Multivariate offset term: Offset term ~ clinical variable of interest + demographic variables

Multivariate isometric term: Isometric term ~ clinical variable of interest + demographic variables

Multivariate shape term: Shape term ~ clinical variable of interest + demographic variables

We note that cerebral parenteral nutrition and ventilation were only assessed in preterm infants and infants with CHD in this study did not undergo prolonged ventilation or parenteral nutrition before surgery, and cerebral oxygen delivery was not available in preterm infants

In table S6, the association between scaling relationship and multivariate morphological term deviance z-scores, calculated using previously reported methods, predict cognitive language and motor abilities in typically developing infants, assessed using the following regression models:

Cognitive/language/motor composite scores ~ deviance z-scores + covariates

In table S7, how whole brain cortical metrics (total surface area, cortical thickness, gyrification index and supratentorial volume) differed between groups was assessed using the following model:

whole brain cortical metric ~ Group + covariates

In table S8, the association between whole brain cortical metrics (total surface area, cortical thickness, gyrification index and supratentorial volume) and cognitive, language and motor scores in each group (controls, preterm infants, infants with CHD assessed separately), using the following model:

Cognitive/language/motor composite scores ~ whole brain cortical metric + covariates
